# Phage Therapy in Plant Disease Management: 110 Years of History, Current Challenges, and Future Trends

**DOI:** 10.3390/plants15030368

**Published:** 2026-01-24

**Authors:** Botond Zsombor Pertics, Lóránt Király, Zoltán Bozsó, Dániel Krüzselyi, Judit Kolozsváriné Nagy, András Künstler, Ferenc Samu, Ildikó Schwarczinger

**Affiliations:** Plant Protection Institute, Hungarian Research Network Centre for Agricultural Research, Fehérvári út 132–144, 1116 Budapest, Hungary; pertics.botond@atk.hun-ren.hu (B.Z.P.); kiraly.lorant@atk.hun-ren.hu (L.K.); kruzselyi.daniel@atk.hun-ren.hu (D.K.); nagy.judit@atk.hun-ren.hu (J.K.N.); kunstler.andras@atk.hun-ren.hu (A.K.); samu.ferenc@atk.hun-ren.hu (F.S.)

**Keywords:** bacteriophage, phage, phage therapy, biocontrol, phytopathogenic bacteria, plant disease management

## Abstract

Bacteriophages, or phages, are viruses that specifically infect and lyse bacterial cells. Since their discovery 110 years ago, they have held a unique place in microbiology, medicine, and agriculture as both scientific tools and potential therapeutic agents. The concept of employing phages to combat bacterial infections, known as phage therapy, predates the antibiotic era and has undergone cycles of enthusiasm, neglect, and revival. Initially explored in the early 20th century, phage therapy offered a targeted biological approach to bacterial disease control. However, the widespread adoption of antibiotics led to a significant reduction in phage research, which only regained momentum in recent decades owing to the global rise of antibiotic-resistant bacteria and increasing demand for environmentally sustainable disease management strategies. This review traces the complete timeline of this history, highlighting key milestones in phage discovery, molecular microbiology, the antibiotic era, and the resulting critical events that spurred the modern phage renaissance in plant disease management. Finally, the significance of cutting-edge integration of synthetic biology, advanced phage delivery systems, and artificial intelligence (AI), which could drive the development of next-generation biopesticides, is also discussed.

## 1. A Short History of Bacteriophage Discovery

This review provides an overview of the historical and scientific milestones in bacteriophage discovery and the application of phage therapy in agriculture, from its discovery and early applications to its industrialisation, decline, and contemporary resurgence. Key chronological developments across both fundamental virology and agricultural applications are shown in Table 1, highlighting the long-standing “dual-track” history and recent convergence that defines the modern era of phage biocontrol.

### 1.1. The Foundations of Lytic Control

Bacteriophages are viruses that target and destroy bacterial cells. Their utility as biocontrol agents for bacterial plant pathogens can be traced back to fundamental discoveries made in human medicine and microbiology. The initial observation of filterable, lytic agents, first by the bacteriologist and physician F. W. Twort in 1915 and independently by F. d’Hérelle in 1917, established the concept of bacterial viruses [1,2]. d’Hérelle, who introduced the term “bacteriophage” (from the Greek words βακτήριο (bacteria) and φαγεῖν (to eat)), demonstrated that these agents can only replicate in the presence of specific bacteria [1,2]. His contributions, such as the plaque assay and concepts of “one-step growth” and “burst size,” are fundamental to contemporary virology [3].

### 1.2. Early Clinical Trials and the Birth of Phage Therapy

d’Hérelle not only discovered bacteriophages but also pioneered their medical application. Phage therapy refers to the therapeutic use of bacteriophages capable of infecting and lysing specific bacterial pathogens, with the aim of reducing or eliminating bacterial populations *in vivo*. In 1919, d’Hérelle initiated one of the earliest clinical trials employing phage preparations for the treatment of bacterial infections [4]. However, the first formally documented therapeutic use is widely attributed to R. Bruynoghe and J. Maisin, who in 1921 successfully applied phage preparations to treat staphylococcal skin infections [5]. Later phage preparations were marketed commercially, including products of d’Hérelle’s Paris laboratory, an enterprise ultimately contributing to the foundation of L’Oréal [6]. By the 1940s, the USA pharmaceutical company Eli Lilly manufactured therapeutic phage formulations targeting pathogens such as *Staphylococcus*, *Streptococcus*, and *Escherichia coli* [7].

In collaboration with d’Hérelle, the Georgian physician and bacteriologist G. Eliava founded in 1923 the institution now known as the George Eliava Institute of Bacteriophages, Microbiology and Virology in Tbilisi, Georgia. He played a leading role in advancing bacteriophage research and therapeutic applications throughout the Soviet Union, and the institute became a global centre for phage production and research. By the 1940s, phage-based therapeutics were widely implemented across the Soviet Union; during World War II, phage preparations were administered to Red Army personnel as prophylactic and therapeutic agents in lieu of antibiotics [3]. However, in 1945, a new era appeared with the golden age of antibiotics. Although dismissed by Western medicine, the use of bacteriophages remained a large-scale, successful practice in Poland and the Soviet Union [8]. This ongoing clinical tradition in Eastern Europe is exemplified by the continued availability of products like Pyophage^®^, Klebsiphage^®^, etc. [3,8].

**Table 1:** Parallel timeline of milestones in phage discovery and phage therapy in plant protection.

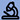 General and Molecular Phage Science and Human Therapy	Year	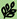 Plant Protection Applications
First observation of phages (Twort) [1]	1915	
Independent discovery and naming “bacteriophage” (d’Hérelle) [2]	1917	
First clinical phage therapy trials (d’Hérelle) [4]	1919	
Founding of Eliava Institute (Eliava & d’Hérelle) [3,9]	1923	
Widespread clinical use (Europe and USSR) [3]	1923–1930s	
	1924	First report of phage activity against a plant pathogen *Bacillus carotovorus* (=*Pectobacterium carotovorum*) [10]
	1925	Coons & Kotila demonstrated that phages could prevent blackleg disease of potato tuber and soft rot of carrot [11]
Discovery of the first antibiotic; penicillin (Fleming) [12]	1928	
	1935	First field trials (reduced Stewart’s wilt on corn) [13]
Phages as essential model systems of molecular biology and biotechnology	* 1938–	
Visualisation by electron microscopy [14,15]	1940	
Nobel Prize (antibiotic) (Fleming, Florey, Chain) [12]	1945	
Hershey–Chase experiment (DNA is the genetic material) [16]	1952	
	1962	First report of streptomycin resistance in *X. vesicatoria* [17]
Nobel Prize (Phage Group: Delbrück, Luria, Hershey) [18]	1969	
	1972	First report of streptomycin resistance in *Erwinia amylovora* in California [19]
First complete phage genome sequenced (ΦX174) [20]	1977	
	1986	First genetic proof of copper resistance in a phytopathogenic bacterium (*X. campestris* pv. *vesicatoria*) shown to be carried by a self-transmissible plasmid [21]
The first unusual repeated sequences, later named CRISPRs, were identified in *E. coli* [22]	1987	
	1989	Invention: host-range mutant (H-mutant) phages patent [23]
	2000	First published field application of the H-mutant phages [24]
	2003	Highlight the need for UV protection of phages [25]
	2005	First EPA-registered phage product (US) (AgriPhage^®^) [26]
CRISPR-Cas proven as anti-phage immunity [27]	2007	Successful utilisation of *Pantoea agglomerans* Eh21-5 as phage carriers in fire blight control [28]
	2012	National/Temporary authorisation in Hungary of ERWIPHAGE™ [29]
	2015	Successful phage biocontrol of Pierce’s disease (PD) in grapevines, caused by *Xylella fastidiosa* subsp. *fastidiosa*, in the USA [30]
First FDA-authorised compassionate use case (USA) (MDR *Acinetobacter baumannii*) [31]	2017	
Engineering phage host-range and suppressing bacterial resistance through phage tail fibre mutagenesis [32]	2019	“Xylencer” project, engineered phages to combat *X. fastidiosa* subsp. *fastidiosa* in olive trees [33]
	2020	Green Deal, Farm to Fork Strategy: The goal is to reduce chemical pesticides by 50% until 2030 [34]
	2021	First EPA registration for *Xylella fastidiosa* phage (on grapevines) XylPhi-PD^®^ [35]
	2024	Engineered “Trojan Horse” phages deliver CRISPR-Cas system to target *Ralstonia solanacearum* virulence genes [36]
	* 2024–	BPSRE (against soft rot) and BAEA (against fire blight) are awaiting full EFSA approval as plant protection products [37]
First effective and viable AI-generated bacteriophage genomes (cocktail of modified ΦX174s vs. *E. coli*) [38]	2025	

* – : from that year

### 1.3. Phage Therapy Eclipsed by the Advent of Antibiotics

The course of phage therapy in the West was radically altered by the advent of antibiotics. A Scottish physician’s (A. Fleming) discovery of penicillin in 1928 and mass production of antibiotics following World War II led to the undisputed dominance of chemical control [12]. Phages were discarded by Western medicine and agriculture in favour of standardised, broad-spectrum antibiotics and pesticides like streptomycin and copper, which were easier to produce, store, and regulate [3].

#### Phages as Tools in Molecular Biology and Biotechnology

Along with their therapeutic application, bacteriophages provided an essential model for the birth of molecular genetics. Beginning in 1938, the Phage Group, led by Delbrück, Luria, and Ellis, utilised phages to study fundamental biological questions concerning mutation and replication [39]. The work of E. M. Lederberg and J. Lederberg led to the crucial discoveries of transduction and lysogeny [40]. The Hershey–Chase experiment, using T2 phages (1952), definitively established DNA as the genetic material [16]. Phages were first directly visualised using electron microscopy by Ruska and Pfankuch & Kausche in 1940 [14,15]. Beyond basic research, phages were integrated into routine microbiology through phage typing for bacterial identification, and their genomes later became indispensable vectors for gene delivery in biotechnology.

**Figure 1:**
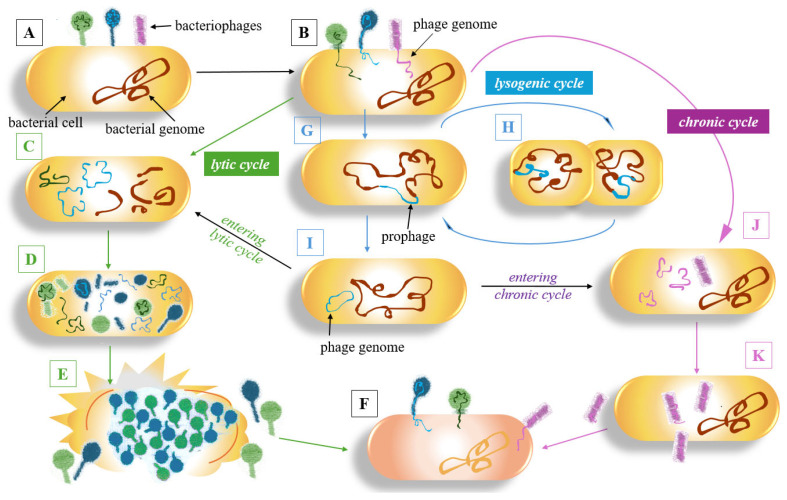
Main steps of phage cycles and mechanisms of bacteriophage infections. Steps of lytic, lysogenic, and chronic infection cycles are indicated in green, blue, and purple, respectively. Phages are coloured green (lytic), blue (temperate) and purple (filamentous). At first, the bacteriophages adsorb on the surface of the host bacterial cell (**A**), then insert their genome into the cell (**B**). Bacterial genome degradation and phage genome replication are performed in the bacterial host (**C**), followed by phage protein biosynthesis, bacteriophage assembly (**D**), host cell lysis (**E**), and release of phage progeny able to infect new cells (**F**). Following the lysogenic cycle, after genome injection, the phage genome integrates into the host genome as a prophage (**G**), which is copied and transmitted to daughter cells after the bacterium replicates (**H**). When exposed to environmental stress, the prophage may be excised from the host genome (**I**), activating the lytic or chronic cycle. During the chronic cycle, phage genome replication and phage protein biosynthesis occur in the bacterial host (**J**), and the newly assembled bacteriophages continuously escape from the intact host cell (**K**) to seek a new host (**F**) [41,42,43].

To fully understand the ecological roles and therapeutic potential of phages, it is necessary to study their reproductive strategies. Generally, bacteriophages are classified based on their relationship with the host bacterium, exhibiting one of three distinct life cycles: the lytic, lysogenic, or chronic cycle (Figure 1).

### 1.4. The Age of Necessity: The Crisis of Antibiotic Resistance Drives Phage Revival

The escalation of antibiotic resistance in bacteria reignited a global interest in phage therapy in the late 20th century. In 2019, antimicrobial resistance was associated with an estimated 4.95 million deaths worldwide, including 1.27 million directly attributable to drug-resistant infections [44]. The economic burden in the USA alone exceeds USD 20 billion annually [45]. Phage therapy offers a highly specific, self-amplifying, and environmentally benign alternative to antibiotics. Recent successful compassionate use cases and clinical trials have been reported across Europe, North America, and Australia. For example, Schooley et al. [31] described the development and successful use of a personalised bacteriophage therapy for a 68-year-old patient with an MDR *Acinetobacter baumannii* infection that did not respond to antibiotics. Two laboratories identified effective lytic phages and formulated therapeutic cocktails under an eIND authorisation, leading to marked clinical improvement and clearance of the infection. Numerous prominent phage centres have been established worldwide, e.g., the Queen Astrid Military Hospital (Belgium), Israeli Phage Therapy Centre (Jerusalem), University of Helsinki, Université de Lyon, and Phage Australia. In parallel, national and international phage banks, such as the Israeli Phage Bank (IPB) and the National Collection of Type Cultures (NCTC) Phage Collection, have been established to ensure access to well-characterised phage libraries. PhageEU is a Brussels-based coalition established in July 2024 by Proteon Pharmaceuticals, JAFRAL, and PTC Phage Technology Centre to represent the European phage community. Its goal is to improve access to phage technology in the EU and shape supportive political and regulatory frameworks [46].

## 2. The Present of Phage Therapy Beyond Medicine: Agriculture Applications

### 2.1. The First Successful Experiments

The idea of using bacteriophages in plant protection is almost as old as the discovery of phages. Parallel to human medicine, research on phage application in plant protection was also initiated. Bacteriophages were first found to be associated with plant-pathogenic bacteria in 1924, when Mallmann and Hemstreet demonstrated that the filtrate of decomposed cabbage inhibited *in vitro* growth of the cabbage rot pathogen *Bacillus carotovorus* (=*Pectobacterium carotovorum*) [10]. Coons and Kotila [11] demonstrated that phages prevent black leg on potato tuber and soft rot of carrot slices caused by *Bacillus atroseptucus* (=*Pectobacterium atrosepticum*) (*Pa*) and *P. carotovorum* subsp. *carotovorum* (*Pcc*), respectively [11]. It is also worth mentioning that Gerretsen et al. [47] first documented the presence of phages in legume root nodules, demonstrating their ability to infect and lyse nitrogen-fixing *Rhizobium* bacteria. In 1934, Massey [48] suggested that the presence of phages in the Nile River was a prime factor in limiting bacterial blight disease in field-grown cotton. The first recorded field trial of plant phage therapy was conducted by Thomas [13], who used phage-treated seeds to successfully control Stewart’s wilt disease in maize, reducing disease incidence from 18% to 1.4%. Phage therapy was tested to control *Xanthomonas*-associated bacterial spot of peaches [49,50], fire blight, and soft rot caused by *Erwinia* species [51,52]. However, initial successes were followed by failures and scepticism by the 1960s [53,54].

### 2.2. Antibiotic and Copper Resistance: Renewed Interest in Using Phage Therapy to Control Plant Pathogens

The widespread use of antibiotics temporally suppressed the use of phages in plant protection, but antibiotic resistance has emerged among phytopathogenic bacteria, as in clinical medicine. Since the 1950s, the extensive use of streptomycin in crops led to the emergence of resistant bacterial strains. Consequently, the use of antibiotics for plant protection is prohibited in EU member states [55]. Copper-based pesticides, introduced in the 1880s, are still widely used for the control of bacterial plant diseases, although the EU has set a target to reduce the use of these harmful pesticides by 50% by 2030 within the framework of the Farm to Fork Strategy and European Green Deal. This is driven not only by the emergence of copper-resistant bacteria but also by concerns regarding copper accumulation in soils. The widespread and often prophylactic use of antibiotics and copper in agriculture eventually resulted in the development of streptomycin-resistant strains of key pathogens like *Xanthomonas vesicatoria* [17] and *Erwinia amylovora* [56]. Genetic evidence emerged that copper resistance in *Xanthomonas* spp. was carried by self-transmitting plasmids [21]. This provided an explanation for the rapid spread of resistance and created an urgent need for alternative biocontrol strategies, which ultimately led to a modern revival of phages in crop protection.

### 2.3. Revived Phage Research

The modern phage revival in agriculture was marked by a critical conceptual breakthrough, the patenting of host-range mutant (H-mutant) phages in 1989 [23], which were selected for a broader host range, allowing them to lyse strains resistant to the parent phage. Flaherty et al. (2000) demonstrated that a mixture of H-mutant phages can successfully control *Xanthomonas campestris* pv. *vesicatoria* (*Xcv*), as well as *X. campestris* pv. *pelargonii* [24]. This research culminated in the first major commercial success: the approval of AgriPhage^®^ in the USA in 2005 for the control of pathogen targets like *Xcv* and *Pseudomonas syringae* pv. *tomato* (*Pst*) in peppers and tomatoes. AgriPhage^®^, developed by Omnilytics Inc. (Sandy, UT, USA), thus became a landmark product, signalling the transition of phage therapy from the laboratory to commercial agriculture. Later field trials demonstrated the practical necessity of this approach [57].

#### Successful Phage-Based Defence Against Bacterial Diseases of Horticultural and Field Crops

Bacteriophages are promising tools for biocontrol applications to manage numerous plant bacterial diseases in different horticultural and field crops. The following section discusses relevant studies on the topic published in the past 15–20 years (for a more detailed summary, see Supplementary Material S2 and S3). Impressive results include demonstrating that a combined or individual application of *Myoviridae* phages derived from soil samples could prevent tissue maceration in bacteria-infected potato tubers by up to 70% [58,59]. Also, phage-treated tomato plants did not display any bacterial wilt symptoms and active (infective) phages could be recovered from roots of treated plants and surrounding soils even 4 months after infection [60]. Mixtures of *Podoviridae* phages could reduce bacterial titres on kiwi leaves 24 h after bacterial infection by at least 75% as compared with untreated plants [61].

In potato cultivation, phage therapy has targeted soft rot and blackleg (caused by *Pectobacterium* and *Dickeya* spp.). A critical comparison reveals that phage cocktails are consistently more robust than monotherapy. Carstens et al. [62] demonstrated that a six-phage cocktail reduced disease incidence by 61% under storage conditions, while Kmoch et al. [63] highlighted a crucial distinction between application timings: preventive treatment gave significantly better results (86.7% efficacy) than curative attempts (54.6%). This suggests that the establishment of the phage population prior to high bacterial loading is vital for success. The above examples also point to the potential of phage therapy beyond field/greenhouse applications: the control of storage diseases caused by pectolytic bacteria (for two recent reviews, see [64,65]). In fact, phage applications may decrease pesticide dependence during fruit and vegetable storage and transport, a particularly relevant aspect for crops with strict residue limits.

In tomato systems, the focus remains on bacterial wilt (*Ralstonia solanacearum*). While traditional cocktails achieve significant reduction up to 80% [66], the field is shifting toward precision tools. Peng et al. [36] introduced an engineered “Trojan horse” phage delivery system using CRISPR-Cas12f1 to disarm virulence genes. This represents a paradigm shift from purely lytic biocontrol to genetic “disarming,” offering a potential solution to the rapid emergence of phage resistance.

The management of *E. amylovora* in apple and pear shows the importance of delivery vehicles. Boulé et al. [67] utilised *Pantoea agglomerans* as a carrier microorganism, achieving up to 96% disease reduction in blossoms. This symbiotic approach addresses the environmental instability of phages. More recent studies, such as Gdanetz et al. [68] and Vique et al. [69], emphasise the necessity of UV protectors and cocktail diversity, showing efficacy (68–82%) comparable with traditional antibiotics like streptomycin. Furthermore, Born et al. [70] demonstrated that engineering phages to overproduce depolymerases can enhance biofilm penetration, increasing bacterial titre reduction to 95%.

For citrus canker (*Xanthomonas citri*) and Pierce’s disease (*Xylella fastidiosa* subsp. *fastidiosa*, *Xff*), new application strategies have been tested. In citrus, combining phages with plant defence activators like acibenzolar-S-methyl (ASM) yielded better results (86% disease reduction) than phages alone [71]. In grapevines, Das et al. [30] proved that phages could move systemically within the plant host to stop progression of *X. fastidiosa*. However, the XYLENCER project [33] took this further by using engineered phages to trigger the plant’s own immune response (PAMP-triggered immunity), merging biocontrol with molecular breeding concepts.

Potential critical factors affecting effectiveness are the following: Concentration (multiplicity of infection, MOI): High titres (typically 10^8^ to 10^10^ PFU/mL) are required for significant field impact. Kmoch [63] explicitly noted that efficacy is dose-dependent; Synergy with conventional treatments: Phages often work best when integrated with other control measures. However, as noted by Balogh et al. [72], the integration with copper-based bactericides can sometimes be less effective than copper alone if the chemical degrades viral particles; Environmental stability: The transition from lab to field remains the greatest hurdle. The use of formulations (sucrose, skim milk, or UV shields) is now considered a requirement for foliar applications [25,68]. Current research indicates that bacteriophages are a viable alternative to antibiotics, particularly in preventative and storage contexts. The trend is moving away from simple isolation and application toward genetically modified phages and integrated pest management (IPM) frameworks. While cocktails provide immediate protection, engineered “Trojan horse” systems and depolymerase-enhanced phages represent the future of stable, long-term agricultural biocontrol.

However, the possibility of comparing the efficacy of phages investigated in different studies is limited. One reason for this limitation is the heterogeneity of strategies (e.g., application methods, phage protection, etc.) and experimental conditions. Furthermore, caution should be taken when assuming correlation between *in vitro* and field efficacy results, as this process should be based directly on the pathosystem [73,74].Therefore, there is a need for generally accepted protocols that would allow for the reliable evaluation and comparison of phage efficacy against specific phytopathogenic bacteria within the plant environment [75].

### 2.4. Phage-Based Commercial Biocontrol Products for Use in Horticulture

As seen from the above (see also Supplementary Materials S2 and S3), there are numerous examples that show a promising perspective for phage applications in horticulture and field crops. However, the implementation of these biocontrol measures in everyday pest management programs can be likely achieved by the application of phage-based, commercial biocontrol products. As a first step towards commercial phage applications in crops, bacteriophage H-mutants were discovered in the 1980s. These phage mutants have a wider host range than wild-type viruses and can kill several strains of plant-pathogenic *Pseudomonas syringae* pathovars, including strains that are resistant to wild-type parent phages. In 1989, the research of L.R.E. Jackson and co-workers led to a patent describing mixtures of these host-range mutant phages that can prevent bacterial plant diseases [23].

#### 2.4.1. Commercial Phage Products That Are Registered (USA, China)

The first commercial phage products registered were those of the AgriPhage^®^ product line (developed by Omnilytics Inc.), which received regulatory approval as biopesticides in 2005 by the US Environmental Protection Agency (EPA). The first AgriPhage^®^ product targeted bacteria causing spot and speck disease in tomatoes and peppers (*Xcv* and *P. syringae* pv. *tomato*). Indeed, this was a milestone marking the transition of phage therapy from laboratory and field trials to a commercial agricultural product. More recently, the company has expanded its portfolio to include additional phage-based biopesticides (Table 2).

Regarding phage composition and symptom reduction provided by AgriPhage^®^ products, it is known that preliminary formulations of the phage cocktail for controlling *Xcv* could reduce disease severity of bacterial spot in tomato by 17% on average [25]. Also, it was found that a phage cocktail against *X. citri* pv. *citri* containing several Omnilytics phages could lower citrus canker disease severity by almost 60% in greenhouse trials [72].

A phage product that the US EPA approved in 2021 as a biopesticide is XylPhi-PD^®^, developed by Otsuka Pharmaceutical against *Xylella fastidiosa* subsp. *fastidiosa* (*Xff*) to protect grapevines against Pierce’s disease (PD). XylPhi-PD^®^ is injected directly into vines with a precision-powered injection device. The company reported that in a field study conducted in California at four different experimental sites, a constant injection of XylPhi-PD^®^ into grapevine xylem vessels reduced PD incidence by 57% [76].

In China, two pesticides containing *Xanthomonas axonopodis* phage YHC5 have been recently approved under registration numbers PD20252172 and PD20252165 [77].

#### 2.4.2. Commercial Phage Products That Are Not Yet Registered (EU)

Although in the USA several phage products have been approved for commercial use in various crops, currently in the EU, no phage-based products have been registered so far by the European Food Safety Authority (EFSA) as plant protection products or biopesticides. There are only four products that, at present, are pending approval: BW-XAJ (bacteriophage against walnut *Xanthomonas arboricola* pv. *juglandis*), BPSRE (bacteriophage of potato soft rot *Enterobacteriaceae*), BACMM (bacteriophages against *Clavibacter michiganensis* subsp. *michiganensis*), and BAEA (bacteriophages against *E. amylovora*) [78]. Nevertheless, in Hungary, a phage cocktail product was authorised for use between 2012 and 2022, initially marketed as ERWIPHAGE, later as ERWIPHAGE FORTE, and most recently as ERWIPHAGE PLUS. The product, which provided effective protection against *E. amylovora* (the causal agent of fire blight in pome fruits), was developed and distributed by the Hungarian company Enviroinvest Ltd. (Pécs, Hungary) [29].

Hungarian authorities granted Enviroinvest permission for the domestic distribution of ERWIPHAGE PLUS. The formulation was updated annually to prevent the development of phage resistance, and its use was restricted to the flowering season; emergency authorisation was granted each year for a 120-day period from mid-March to mid-July [76]. Furthermore, the Scottish company APS Biocontrol LTD (Dundee, Scotland) developed a phage cocktail (Biolyse) as a patented technology for the postharvest treatment of potato tubers to prevent soft rot caused by *Pectobacterium* spp. [37,79,80]. The above-mentioned examples highlight the introduction of phage biocontrol products into the European market, despite existing regulatory hurdles. The commercial and regulatory landscape for phages is highly differentiated, with the USA providing the most established pathway through the EPA, while the EU relies on national or temporary authorisations pending unified EFSA approval.

## 3. Challenges and Mitigation Strategies

### 3.1. Host Range and Resistance

The use of bacteriophages has both advantages and limitations, as compared with other methods of pest management. However, the application of bacteriophages can be a part of a long-term strategy in agriculture, it is environmentally friendly, and it can be integrated well into farmers’ plant protection solutions.

#### 3.1.1. Host Specificity: Blessing or Curse?

Bacteriophages generally display a narrow host range, which has important practical implications for plant disease management. Many phages infect only a single bacterial species or even specific strains within that species; broader host ranges are less common [81,82,83].

A key advantage is the minimal off-target impact on non-pathogenic or beneficial microbiota in the plant environment. By sparing commensal and symbiotic bacteria, phage treatments help preserve the natural microbial community that supports plant health and resilience. At the same time, the high specificity allows effective suppression of target phytopathogens [84,85].

However, the same specificity also represents a major limitation: a single phage may fail to infect all strains or variants of a given pathogen. This strain-level variability can reduce the robustness of phage-based control strategies unless multiple phages are combined. Despite this challenge, the immense natural diversity of bacteriophages means that most bacterial strains are susceptible to at least one phage, providing a broad reservoir for biocontrol agent discovery and optimisation [86].

#### 3.1.2. Impact of Phage Biocontrol on a Given Microbiome

The effectiveness of biological control by phages depends on the specific microbiome into which they are introduced [87,88], and this efficacy can be increased by exploiting beneficial effects of local microbiota [89]. For example, phages, being abundant in soil and especially in the rhizosphere [90], are key components of the soil microbiome, where elements of a given microbial community in a habitat with distinct physicochemical properties are constantly interacting [87]. Therefore, when applying phages, their impact on soil ecosystems must also be considered [37,91,92]. Omics-based methods are ideal to study interactions between the soil microbiome and phages under changing environmental conditions. For example, altitude and soil water content can significantly affect the impact of phages on the soil microbiome [93,94]. Importantly, tomato plants grown in a phage-depleted soil microbiome were more susceptible to infection by *P. syringae* pv. *tomato* as compared with control plants grown in soil with normal phage amounts in the microbiome [95]. Fortuna et al. [96] reported that when using a sensitive method to test the environmental safety of phage biocontrol, *Xcc* phages appeared not to affect non-target species of the soil microbiome while reducing the biomass of their bacterial host. Studying the effects of tomato rhizosphere phage communities on bacterial wilt disease outcomes caused by *R. solanacearum* revealed that *R. solanacearum*-specific phages were more abundant in healthy plant rhizosphere and that phages targeting pathogen-inhibiting bacteria had a stronger impact in the root zone of diseased plants [97]. Phage application by root drench shifted the diversity of the tomato rhizosphere microbiome and resulted in reduced *R. solanacearum* and increased phage densities. Moreover, enriched bacterial taxa in these shifted microbiomes showed control efficacy against *R. solanacearum*, and one of these, *Burkholderia* sp. B12, was able to completely inhibit disease symptoms *in planta* [98]. Repeated application of a phage cocktail with four *R. solanacearum* phages in tomato greenhouse and field trials not only reduced pathogen density but also increased the resident rhizosphere microbiome diversity and specifically enriched Actinobacterial taxa. When tested in greenhouse experiments, these taxa exhibited synergistic pathogen suppression when applied along with the phage cocktail. Wang et al. [89] reported that pathogen density control of a phage cocktail combined with specific *Nocardioides* or *Streptomyces* strains was improved by 55% and 40%, respectively, as compared with phage-only treatments.

The impact of *E. amylovora*-specific phages on the flower microbiome was also recently evaluated. Two consecutive AgriPhage^®^ applications on flowering apple trees were not harmful to the flower microbiome [68], and flower application of phages was found to significantly reduce fire blight symptoms in pear blossoms inoculated with *E. amylovora* without changing the flower microbiome [99].

#### 3.1.3. Bacterial Defence Against Bacteriophages: Phage Resistance

Like other detrimental environmental effects, bacteria can adapt to the presence of bacteriophages. Resistance to phages can rapidly develop in the bacterial population through horizontal gene transfer among susceptible bacteria due to the narrow strain specificity of certain phages, which limits their effectiveness in pathogenic populations with more diverse genomes or different geographical variations. As a result, bacteria can become resistant to phage infection to varying degrees, which may limit the applicability of phage biocontrol methods. Several mechanisms are described to gain resistance, e.g., masking the phage receptor (e.g., overproduction of capsular polysaccharide) [100,101], inhibition of phage DNA injection, phage DNA replication, influencing the phage assembly, degrading phage DNA, and even self-destructing in order to inhibit phage reproduction [102]. The rate of development of resistance can vary greatly depending on the bacterial species, the phage, and the environmental conditions. In laboratory *in vitro* conditions, under high selection pressure, resistance can develop quickly (within hours to day) [103]. However, in *in vivo* or in plant field experiments, the appearance of resistance is significantly slower and may be influenced by different factors [89,104,105]. For example, larger, more diverse bacterial populations increase the chance of resistant mutants arising [106]. In addition, a number of environmental factors, such as UV radiation, temperature, humidity, etc., which affect bacteria, phages, or both, can influence the development of resistance [107,108,109,110,111]. Since resistance has consequences that are, to varying degrees, detrimental to the bacterium (trade-offs), these impose a limit on the development of phage-resistant bacteria in nature. Mutations affecting the surface molecules of bacteria (lipopolysaccharides, extracellular polysaccharides, flagella, and pili), which are responsible for the initial attachment of phages and infection, may also affect the virulence of bacteria, but their loss or modification may be associated with other fitness traits that are not directly related to pathogenicity but may negatively affect the survival of the bacteria in the environment [112,113,114,115,116]. In addition, the likelihood of bacterial resistance developing is lower with phages than with antibiotics, so the development of certain phage preparations can be considered more economical than developing new antibiotics or antibacterial pesticides [89].

#### 3.1.4. Phage Cocktails Can Be Used to Overcome Resistance and Narrow Host Range

Developing a phage biocide that eliminates each strain of a particular bacterial species can be challenging; although, it is known that a single phage can be active against several pathovars of, e.g., *Pseudomonas syringae* pv. *actinidiae* (*Psa*) (causal agent of kiwifruit canker) [117]. In order to create an effective phage cocktail for biocontrol application, various lytic phages should be selected, having diverse proteins that bind different bacterial receptors, to avoid or minimise the development of phage resistance [36,66,67,94]. To further avoid inefficiency and minimise the targeting of beneficial bacteria, the host range and virulence against plant-pathogenic bacteria of each candidate phage should be assessed prior to phage cocktail preparation [118].

The fact that phage cocktails can effectively control plant-pathogenic bacteria has been shown by several studies. Some relevant examples were already presented in the “Successful Phage-Based Defence Against Bacterial Diseases of Horticultural and Field Crops” section for *Dickeya solani* [119], *R. solanacearum* [66,120], *X. fastidiosa* [30], and *E. amylovora* [121]. In a different study, it was reported that a six-phage cocktail reduced incidence and severity of black leg disease of potato stems (caused by *P. atrosepticum*) by more than 60% [62].

Despite the above-mentioned successful applications of efficient phage biocontrol preparations, it should be emphasised that it might not be possible to develop a single, multidimensional phage cocktail that is and remains highly effective towards most or all bacterial phytopathogens—likely due to the complexity of plant–pathogen interactions. In fact, phage cocktail applications require the continuous identification of phage-resistant bacteria emerging over time and the modification of phage cocktail formulations to ensure the effective killing of target bacteria [122]. Regarding commercial phage applications in crops, the AgriPhage^®^ product line is periodically updated by the developing company (Omnilytics Inc.), involving a constant monitoring of phage populations [79]. Also, the phage cocktail ERWIPHAGE PLUS is modified each year to prevent the development of bacterial resistance and sustain the efficiency of biocontrol [29].

Although phages within a cocktail typically act additively or synergistically, cases exist where fewer phages achieve greater efficacy [60,123]. Synergy may arise when one phage facilitates infection by another, for example, a tailspike enzyme removing capsular barriers and improving receptor access [124]. Potential antagonistic interactions can be minimised by selecting a smaller number of phages with broad host specificity, which also simplifies large-scale production [59,125]. Consequently, the optimal cocktail size depends on both phage properties and the diversity of target bacterial populations.

Host-range expansion can be achieved not only by incorporating additional isolates but also by generating host-range mutants (H-mutants). Phage cocktails containing H-mutants have been successfully applied against several plant-pathogenic bacteria [24,25,126]. These mutants are obtained by first isolating phage-resistant bacteria and then selecting spontaneous phage variants capable of infecting both the original and resistant hosts. A related approach, the step-by-step (SBS) method, iteratively isolates resistant bacterial strains and the phages that overcome them [127].

Therefore, in practical applications, it is advantageous to use not a single phage isolate, but a mixture of several isolates. Phage therapies that are commercially available for plant treatments or successfully used in various experiments therefore mainly consist of phage cocktails [79]. The utilisation of phage cocktails can be improved by rotating phage types over time, combining phage treatments with other control methods such as bactericides or plant defence inducers [89].

### 3.2. Stability, Formulation, and Translocation

Since phages can be found where bacterial strains are present, e.g., soil, water bodies, animals, plants, or the human body, isolation is relatively simple, and their storage is especially easy because they are not living organisms, so maintenance is highly cost-effective [128,129]. However, their stability is easily compromised. It can generally be said that their numbers can decrease significantly during storage, which can best be prevented by using the cold chain system. However, even in such cases, their shelf life is significantly shorter than that of other compounds used for chemical treatment [130]. Legal regulation issues of antimicrobial phage products mostly arise from the need for continuous modification due to shelf-life deterioration, phage rotation, loss of effectivity, etc. One major practical limitation to the utilisation of phages is their susceptibility to the exposure to environmental influences. Phages can only reproduce in the presence of their host bacteria and are particularly sensitive to changes in environmental conditions.

#### 3.2.1. Efficacy of Phages from the Perspective of Application Strategies

When using phages as biopesticides, the activity and persistence of phages can be significantly increased by different practical strategies (protective adjuvants, encapsulation, optimised application timing, and/or frequency), thereby making bacteriophage-based plant protection treatments more effective. However, it is also important to consider practical factors that significantly influence the quality of application and marketability of phage preparations. These include application dose and time (for optimal effect), uniform distribution and persistence on the plant surface, compatibility with equipment (applicability with existing sprayers), and consistent treatment intervals. The formulation of phage preparations and the use of additional substances are crucially important. The formulation is not only essential during application, but also important for storage. Currently, there is no known gold-standard procedure that would significantly increase storage time and extend shelf life, either in liquid or dry forms. The formulation essentially plays a role in reducing exposure to environmental factors such as temperature fluctuations, dehydration, and the negative effects of UV radiation.

Several studies demonstrate the significance of spatially and temporally diverse phage applications that conform to the infection strategy of a certain phytopathogenic bacterium in a given crop. A typical example may be the treatment of soft rot disease (caused by *Pectobacterium* spp. or *D. solani*) in potato, where the main focus is on postharvest control in tubers, since this is the time and site of bacterial infection [62,119]. In contrast, the control of soil-borne *R. solanacearum* provides optimal results when phages are applied in soil-based systems [66,120,131]. In fact, soil treatments can also increase viable seedling numbers of watermelon infected by the bacterial fruit blotch pathogen *Acidovorax citrulli* while a preventive phage-based seed treatment may significantly limit the impact of the bacterium on its plant host [132,133]. On the other hand, for diseases manifested at the phyllosphere, including those caused by *P. syringae* pathovars, spray treatments by phages appear to be the best means to reduce bacterial titres and symptom development [61,134,135].

One of the limitations of effective phage biocontrol in crops is the possibility of poor phage persistence on the phyllosphere and rhizosphere due to adverse environmental factors like, e.g., extreme temperatures, desiccation, excess rainwater, UV radiation, and chemicals.

A possible strategy to improve phage survival is the use of an avirulent strain of the target bacterial pathogen or other phage-sensitive but non-pathogenic bacterium species as phage carriers. For example, phages mixed with such bacterial species were used to enhance phage persistence on the leaf surface and were shown to improve biocontrol of black rot disease of broccoli plants (caused by *Xcc*) [136]. This can help the phages survive even if the target bacteria are not present during treatment [137,138].

It is also known that avoiding daylight (UV radiation) during application can indeed improve phage-based biocontrol. Phages are rapidly inactivated by UV radiation, as UV radiation can damage their DNA and inhibit replication. As a result, the UV radiation may reduce their persistence on plant surfaces, so the effectiveness of phage treatment is significantly reduced under intense sunlight [139]. For instance, applying phages to tomato leaves in evening hours resulted in their extended persistence in the phyllosphere, providing more time for phages to infect and kill their bacterial target causing leaf spot, *Xcv* [25]. Indeed, several studies have demonstrated that phage applications after sunset [140] or at dawn can significantly improve phage longevity within the phyllosphere, thereby conferring an extended window for bacterial infection and control [141]. Accordingly, drought also negatively affects the viability of phages, along with temperature extremes that compromise their stability. For this reason, a significant seasonal effect can be observed in the activity of phages throughout the year [142]. Importantly, such strategies demonstrate the significance of optimising the timing of phage applications in order to maximise their efficacy in horticulture and field crops. Light sensitivity can also be mitigated through direct plant injection [131].

#### 3.2.2. Translocation of Phages

One possible approach to increase the effectiveness of phage biocontrol is to deliver the phages into the plant. It has long been known that plant viruses are capable of moving within plants [143]. Phages can enter the plants from the rhizosphere through roots or tubers as well as from the phyllosphere and may translocate to distant parts of the plant through vascular tissues [132,144]. *In planta* systemic movement of phages has been reported in various bacteria—host plant relationships such as in case of *X. oryzae* phages in rice [145], *R. solanacearum*, *X. perforans*, and *X. euvesicatoria* phages in tomato [146], *E. amylovora* specific phages in apple and fire thorn [144], or *A. citrulli* phages in melon [132]. Following penetration, phages may not only remain viable in plant tissues for days [146] or even for months [60] in the absence of host bacteria, but they can also reduce the development of disease symptoms in the presence of their host. Reddy et al. [147] observed that lesion development in rice leaves inoculated with *X. oryzae* was entirely controlled when plants were fed with a phage suspension through roots for 12 h prior to bacterial inoculation. Similarly, Kolozsváriné Nagy et al. [144] reported that *E. amylovora*-specific phages applied to either twenty-week-old apple roots by drenching or to aerial parts of the plants by spraying one day before bacterial inoculation significantly reduced fire blight symptoms. Holtappels et al. [148] tested an irrigation-based phage application by dosing an *Xcc* phage cocktail to cauliflower seedlings repeatedly over a six-week period before bacterial inoculation. Ten days after inoculation, the phage cocktail was able to reduce disease symptoms. Certain phages can also be effective when applied post-inoculation. Soil application of a phage four days after spray inoculation with *A. citrulli* of melon leaves, when symptoms have already developed, significantly diminished disease severity and also halted symptom progress [132].

#### 3.2.3. The Formulation of Bacteriophages for More Effective Plant Protection

Ensuring the stability and preserving the viability of phage preparations during storage and transport is critical, yet challenging, as phages are sensitive to various physical and chemical conditions [149]. Numerous studies have been conducted in recent years using a variety of supplementary compounds, including aromatic amino acids, biodegradable or hydrogel polymers, milk protein (casein), Tween 80, and vegetable-based extracts (beetroot, carrot, paprika), which can play a role in UV protection. These adsorbent matrices can protect the virions from UV radiation and desiccation, thereby extending their persistence on leaf surfaces [25,130,150,151].

Experiments were also conducted related to formulation development, which improved the effectiveness of phage treatment. These were generally multicomponent, but, for example, when bacteriophages were used together with acibenzolar-S-methyl (ASM), it protected tomato plants with significantly greater effectiveness than the phage alone [152]. Another advantage of formulation is its ability to increase storage stability, which ultimately also increases the reliability of the future product. Cryoprotectants (e.g., glycerol) are primarily used for this purpose and are used along with a freeze-drying process, but antioxidants have also been added in some cases to prevent the inactivation of phages by oxidative damage [153]. Too high or low pH-values can also reduce phage viability, which may affect their combined use with various agrochemicals. Most agrochemicals are slightly alkaline, but phages have varying pH sensitivities depending on the surface environment. Phages are typically most sensitive when the air temperature is above 37 °C, the pH value is below 5, and they are also exposed to UV radiation [130]. The use of pH buffers also increases the viability of phages during storage and application by maintaining a favourable pH environment [154].

Encapsulation is also a promising approach, which makes phages less susceptible to various environmental conditions while allowing for controlled release from the capsule. Two approaches have emerged in this regard: one involves phages packaged in liposomes (mainly medical and veterinary applications), while the other involves the use of polymer-based microcapsules [155]. The latter is much more popular as its production does not always require a specialised laboratory infrastructure. Nanoparticles provide highly sophisticated and versatile systems that can be used to transport phages and simultaneously deliver other complementary substances, thus enabling the development of complex solutions, such as the simultaneous delivery of phages and antibiotics with controlled distribution [156].

An innovative approach to enhance bacteriophage stability is the application of nanomaterials as phage carriers. Nanomaterials typically have a particle size between 1 and 100 nm. Remarkably, it was shown that a nano-N–acetylcysteine–zinc sulphide (nano-NAC-ZnS) formulated phage (ΦXp06-02-1) displays a significantly improved persistence in UV light both *in vitro* and in the phyllosphere. In fact, after eight hours of sunlight exposure, bacteriophage persistence was 15-fold higher in the phyllosphere of tomato plants when formulated with nano-NAC-ZnS, as compared with non-formulated phages. Furthermore, nano-NAC-ZnS exerted a bactericide effect against two strains of *X. euvesicatoria* pv. *perforans*, causal agents of bacterial leaf spot, even after 24 h of incubation [157].

#### 3.2.4. Efficacy of Phages Compared with Antimicrobials and Other Biocontrol Agents

When comparing the use of phages with other chemical or biological methods, several factors justify their use. Importantly, these viruses are not dangerous to eukaryotic organisms, meaning they are not harmful to animals, plants, or humans [158]. Phage populations increase through replication when host bacteria are present, unlike chemical treatments, whose concentrations decrease over time. After elimination of bacteria, inactivated bacteriophages decompose and return to the natural organic cycle. It is notable that phages are a natural part of the agroecosystem; therefore, changes in their quantity do not pose any risk. Most bacteriophages are not included in food chains; thus, they are not capable of accumulation [158,159].

In past decades, chemicals (pesticides) have been widely used to protect crops against bacterial diseases. As a consequence, several phytopathogenic bacteria have developed resistance to, e.g., copper-based pesticides, hydrogen peroxide, and other chemicals including antibiotics. Due to this fact and human health and environmental concerns, the restriction and replacement of these chemicals—at least partially—often emerges as a public and governmental issue. In this regard, it is of pivotal importance whether bacteriophage applications can be as efficient as, e.g., antibiotic or copper-based treatments?

Based on literature data, it seems that, at least in numerous cases, phage treatments may provide antibacterial effects comparable with those of chemical, e.g., antibiotic treatments. For example, it has been demonstrated already in 2011 that the phage ΦEa2345-6 (with the epiphytic bacterium *P. agglomerans* Eh21-5 as a carrier) suppressed *E. amylovora* infection on flowers of potted apple trees with an effect comparable with that of the antibiotic streptomycin [67]. Also, injection of the stem of one-year old pear plants with a single phage resulted in the absence of fire blight symptoms to a similar degree as in plants that were treated with antibiotics [160]. Pre-treatments by a cocktail of two phages or individual phages significantly reduced symptoms of soft rot (*Pectobacterium* spp.) by 60 to 95% in Chinese cabbage detached mature leaves, and the phage cocktail was as effective as commercial antibiotics. The pre-treatments in seedlings, however, reduced the severity of symptoms but were not as effective as antibiotics [161].

Regarding the efficacy of bacteriophage treatments vs. copper-based chemicals, Flaherty et al. [24] investigated cocktails of H-mutant bacteriophages for the control of bacterial spot (*Xcv*) in tomato. The phage-treated plants (both in greenhouse and field conditions) displayed more than 17 to 25% disease severity reduction and an almost 24% fruit yield increase, results superior to those obtained with copper-based traditional pesticides. In other field experiments with tomato, a formulation of six different phages specific to *Xcv* race T3 strain 91–118 was used. Applications of these phages were effective against the bacterial spot pathogen in tomato, showing a better disease containment than with copper–mancozeb treatments or in untreated controls [162]. Also, field trials showed that weekly and biweekly applications of a phage mixture (AgriPhage^®^) reduced disease severity of *Xanthomonas* leaf blight in onion (caused by *X. axonopodis* pv. *allii*) in an equivalent or better manner than weekly applications of copper hydroxide plus mancozeb [163]. Furthermore, in an experimental citrus nursery, phage applications significantly reduced the progress of citrus bacterial spot (caused by *X. axonopodis* pv. *citrumelo*) in moderately susceptible Valencia oranges, providing similar levels of control to copper–mancozeb treatments [72].

The efficacy of bacteriophage treatments can be often similar to antibacterial protection conferred by certain biocontrol agents, including several species of soil-borne, non-pathogenic fungi and bacteria. Soil-borne fungi that belong to the *Trichoderma* genus display broad-spectrum antagonistic activities towards diverse types of plant pathogens, including bacteria. In fact, the effect of *Trichoderma*-based biocontrol methods towards plant-pathogenic bacteria can be comparable with that provided by phage applications. For example, *T. harzianum* produces several antibacterial secondary metabolites (e.g., viridiofungin, trichokonin and lysozyme) highly effective towards bacterial plant pathogens like *C. michiganensis* and *E. amylovora*. Also, a crude extract of *T. harzianum* inhibited the growth of *R. solanacearum* (causal agent of bacterial wilt) both *in vitro* and *in planta* (on tomato), and scanning electron microscopy confirmed the disruption of bacterial cells. Furthermore, field applications of *T. asperellum* isolates T4 and T8 in tomato significantly delayed the onset of bacterial wilt while improving plant growth and yield [76].

Certain soil-borne, plant growth-promoting rhizobacteria (PGPR) emit volatile organic compounds (VOCs, e.g., 3-hydroxy-2-butanone and acetoin, 2,3-butadial) that may efficiently restrict the growth of plant-pathogenic bacteria. For example, it has been shown that *Pseudomonas fluorescens* WR-1 produces VOCs that significantly suppress *R. solanacearum*-caused bacterial wilt in tomato. These VOCs could decrease exopolysaccharide (EPS) production, biofilm formation and root colonisation by *R. solanacearum* by 42%, 49% and 35%, respectively [164]. In a similar study, two isolates of *Bacillus velezensis* (Y6 and F7) could suppress the incidence of wilt disease in tomatoes by ca. 50% [165]. Importantly, these efficiencies for controlling bacterial wilt by PGPR are comparable with that conferred by phage treatments (20–80%) [89,166].

#### 3.2.5. Versatility: Synergistic Effect of Phages

Nevertheless, overall effectiveness of bacteriophages is comparable with that of antibiotics, if not more convincing, most importantly, due to the ability of penetrating biofilm matrices, which can significantly reduce the rate of biofilm formation on the surface of various plant parts [159]. Repressing biofilm formation and degrading existing biofilms highlight exploiting the synergy of different treatments based on using phages in combination not only with other different phages in a cocktail, but also with various antibacterial compounds [167].

The inclusion of various bacteriophages in plant protection strategies is relatively easy to implement, as they can be incorporated into many integrated pest management systems and used as a supplement to various biological and chemical solutions [168]. As a result, the combined plant protection protocol requires less pesticide when using bacteriophages, which is not only environmentally friendly but also increases cost-effectiveness [169]. In integrated systems, the chance of bacterial resistance developing can also be minimised, as the antibiotics usually target essential bacterial processes, while bacteriophages aim to bacterial surface receptors to gain entry into the bacterial cell [170]. This year, it has come to light that certain bacteria can develop resistance simultaneously with the help of their integron cassettes [171], making the use or combination of phages increasingly inevitable in order to have the appropriate tools for treating bacterial infections [172].

## 4. Safety and Regulation

### 4.1. Authorisation

The use of bacteriophages enables the creation of promising and potentially integrated biological control strategies for treating various diseases caused by plant-pathogenic bacteria. Besides the previously mentioned challenges (e.g., long-term stability in the environment, broadening their narrow host range), harmonising and standardising the regulatory frameworks must also be addressed. Thus, bacteriophage-based applications should become available globally as a commercial plant protection product, rather than products that can only be marketed under restrictive regulations.

The development of regulatory approval processes, standardised protocols, and regulatory pathways is a key area of successful phage-based biocontrol applications. Collaboration between researchers, industry, and regulatory authorities is necessary to establish clear guidelines for phage manufacturing and quality control. Harmonising regulations internationally would further facilitate broader development and access. Modern research, leveraging the tools of genomics and synthetic biology, is paving the way for more effective, reliable, and safer phage-based therapies [173]. A surge in global interest was spurred by the successful FDA-authorised compassionate use of personalised phage cocktails in the USA in 2017 to cure a patient with a MDR infection [31]. This high-profile medical success lent credibility and urgency to agricultural research. Simultaneously, European commercialisation began to take hold through local, often temporary, authorisations (c. 2018). Countries like Hungary saw products like ERWIPHAGE PLUS™ enter the market to combat fire blight, demonstrating that despite the lack of a unified EU-wide approval (which remains a challenge), the regional demand for non-antibiotic solutions was driving uptake.

At present, the widespread use of clinical phage therapy [174,175] and phage technologies in the food industry [176] are differentially regulated worldwide. The same applies to pesticides; different countries apply specific regulatory processes in accordance with local authorities [177].

### 4.2. Regulations of Phage-Based Biopesticides

Despite increasing research and compelling results, only a limited number of phage-based biopesticides are available worldwide [37], likely due to authorisation procedures being subjected to various regulatory requirements that are often different from country to country [178] or the lack of guidelines specifically developed for bacteriophages [137]. In the USA, the Environmental Protection Agency (EPA) regulates and authorises bacteriophage-based biopesticides, which are exempt from tolerance for residues [137]. In China, the Institute for the Control of Agrochemicals (ICAMA) under the Ministry of Agriculture and Rural Affairs (MARA) is the registration authority for pesticides including biopesticides [177]. The institute, according to the relevant Regulation on Pesticide Administration, is responsible for various tasks, e.g., national pesticide registration, evaluation, surveillance, monitoring risks of pesticide application, and import/export management [77], cooperating closely with international organisations. In the USA, the Biopesticides and Pollution Prevention Division (BPPD) of the EPA regulates and authorises bacteriophage-based biopesticides [137,179]. Moreover, in the USA, biopesticide registration requires less data and time compared with conventional pesticides, thereby promoting the use of lower-risk plant protection products. However, in the EU, the strict regulation referring to the authorisation, sale, and use of plant protection products (Regulation (EC) No 1107/2009, updated in 2024) by the European Agency for Safety and Health at Work (EU-OSHA) does not specifically address bacteriophages [180]. Whereas phage products must be regularly updated to ensure effective protection against emerging bacterial strains, any change in a composition of a phage cocktail requires a new registration at the EU level, which involves considerable time and costs [137]. Therefore, there is a strong need for clear, comprehensive, and standardised guidelines for using phages in agriculture [96,178]. The WHO Regional Office for Europe along with the Global Antimicrobial Resistance Research and Development Hub are also fostering phage applications [181], just as the EU is determined in setting up better and harmonised quality, safety, and efficacy protocols [182]. Regarding the application of phages in the food industry and agriculture in the USA, a variety of recommendations have been recently reported [178], partly as regulatory needs, including clear guidelines, post-commercialisation monitoring, and standardised labelling; and also as research needs, such as fostering public–private collaboration, enhancing production methods and efficacy, and integrating phage applications into sustainable practices [137,182].

### 4.3. The Phage Genome Menace

Manufacturing and quality control of phage preparations present distinct technical challenges. Producing well-characterised, high-titre phages that are free from bacterial debris, endotoxins, and potentially harmful genes requires advanced purification and rigorous quality assessment techniques. Historically, insufficiently purified phage suspensions were a major source of problems and mistrust toward phage therapy in the 1930s [149]. Even if we produce a de facto clear phage suspension, the theoretically harmless phage particle bears additional potential perils inside.

One of the main generic concerns with using naturally occurring, untailored phages for any therapy lies in their different modes of the reproduction cycle and is associated with the risk of horizontal gene transfer via genetic material and the presence of hypothetical phage proteins with unknown functions.

The traditional phage families (which were abolished as taxonomic groups in 2023, [183]) tend to follow one or the other route of the phage infection cycle (Figure 1). Myoviruses (contractile, non-flexible tail, e.g., phage T4) usually have a broad host range, are obligate lytic (virulent), and employ the lytic phage cycle, which eventually leads to the lysis of the infected bacterium and the release of viruliferous phage progeny (Figure 1A–F); they are potentially convenient for therapy [184,185,186,187,188,189].

Siphoviruses (characterised by a flexible, non-contractile tail, e.g., phage λ) are often temperate phages with narrow host range capable of making a lysogenic decision by integrating their genome into the bacterial chromosome (lysogeny), and they replicate as a prophage during host cell division (Figure 1G,H) [159,170,182].

**Figure 2:**
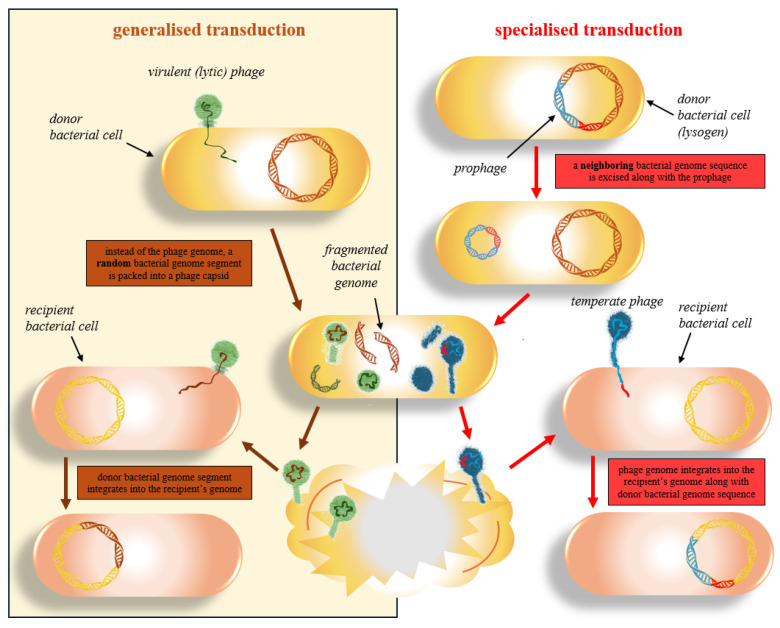
The process of generalised (**left**) and specialised (**right**) transduction. Steps of the two processes are represented by brown and red arrows (and text boxes), respectively. Genome colouring: green—virulent phage genome; blue—temperate phage genome; brown—donor bacterial genome; yellow—recipient bacterial genome; red—neighbouring donor bacterial gene.

Concerns arise regarding their therapeutic application:

(i) They do not necessarily cause lysis;
(ii) They can confer various advantages to their bacterial hosts through specialised transduction (Figure 2);
(iii) “Silent” prophages residing in the bacterial genome may protect the bacterium from subsequent infections by other phages (superinfection exclusion) [185,186,190,191,192,193].

Generalised transduction (Figure 2) can also occur in obligate lytic phages. Such phages may also act as vectors for mobile genetic elements, including antibiotic resistance genes [194]. The solution to this issue is the genetic engineering of phages or the use of recombinant phage enzymes, which are discussed below.

## 5. The Dawn of Designed Phages: Engineering the Next Generation of Antimicrobials

The contemporary era of bacteriophage (phage) research is characterised by a definitive shift from the isolation of naturally occurring phages to their purposeful genetic engineering. Initially, phage therapy relied on collecting suitable lytic phages and utilising empirical methods, such as preparing phage cocktails or selecting host-range mutant phages, to address resistance and broaden efficacy [195]. However, advances in genomics, molecular biology, and synthetic biology now make it possible to artificially modify phages to enhance their therapeutic performance, also solving the limitations of conventional phage therapy due to the ambiguousness of the phage’s genetic material; thus, augmented safety could be achieved.

A critical foundational step was the sequencing of the first phage genome, PhiX174, in 1977 [20], which established the blueprint for modern safety screening, ensuring that therapeutic preparations are free from virulence or toxin genes. Furthermore, understanding fundamental bacterial defences, such as the confirmation that CRISPR-Cas systems function as an anti-phage adaptive immune mechanism [27], has directly informed the design of robust phage countermeasures.

A growing interest surrounds the application of synthetic, genome-designed phages in both medicine and agriculture. Reflecting this trend, the World Economic Forum (WEF) ranked such phages among the most promising emerging technologies of 2023, highlighting their potential not only in human therapeutics but also in the biocontrol of phytopathogenic bacteria, where they have shown remarkable effectiveness [36].

### 5.1. Engineering for Enhanced Efficacy

Phage engineering now provides the ultimate tools to overcome microbial resistance. Foundational work in synthetic phage engineering demonstrated that phage host range can be systematically broadened by modifying tail fibres [32], allowing researchers to custom design a phage to defeat multiple resistant bacterial strains. This approach is analogous to antibody engineering, creating specific “phagebodies.” In the case of such chimeric phages, beneficial structural or functional proteins from different phages are combined to create recombinant particles with enhanced properties, such as increased biofilm penetration or the ability to target intracellular pathogens [196]. Phages can also be modified to act as delivery vehicles to introduce various agents into bacterial cells (e.g., RNA molecules, transcriptional regulators), or their protein coats can be decorated with functional molecules such as antibiotics.

If a temperate phage otherwise meets all therapeutic requirements, its genome can be modified to convert it into an obligately lytic variant, for example, by deleting the lysogeny module, such as the *integrase* gene. *In vivo* studies have demonstrated that even temperate *Staphylococcus* siphoviruses can be effective against MRSA strains [197,198], including mutants with a disrupted lysogeny module [199,200].

To enhance the biosafety of lytic phages, the likelihood of generalised transduction can be reduced by designing a phage genome so large that it physically limits the incorporation of additional DNA fragments into the capsid. However, constructing such recombinants with increased genome length may encounter packaging constraints of the phage capsid, thus requiring the identification and removal of nonessential genes. Such genes, encoding hypothetical or uncharacterised phage proteins, can also be selectively removed and replaced with genes encoding proteins of known beneficial function, for instance, enzymes that accelerate degradation of bacterial DNA during infection, thereby reducing the chance of bacterial gene packaging into the capsid. The integration of CRISPR-Cas9 gene-editing techniques allows phages to be repurposed this way, offering a powerful strategy against antibiotic-resistant bacteria [201]. A recent study showcased this potential in agriculture, where an engineered filamentous phage was used as a “Trojan horse” to deliver a CRISPR-AsCas12f1 system to disarm the virulence gene hrpB in the plant pathogen *Ralstonia solanacearum*, effectively controlling bacterial wilt in both laboratory assays and plants [36]. Filamentous phages (e.g., *M13*) can follow an alternative infection pathway (chronic cycle), being continuously secreted from the host cell without inducing lysis (Figure 1J,K) [122]. The principle of phage display technology relies on the ability of filamentous phages to physically link a target protein expressed on their capsid to the corresponding gene sequence that encodes it, thereby enabling the direct identification of an unknown genotype from its displayed phenotype. This powerful approach has been successfully employed in various biotechnological and medical applications, including the selection of SARS-CoV-2-specific antibodies [202]. Phage display has revolutionised the creation of extensive libraries of genetically modified phages; the enormous diversity makes it possible to rapidly and efficiently identify the phages most suitable for therapeutic applications [203]. Techniques such as biopanning, involving repeated cycles of selection and amplification, are crucial for enriching phage clones with high binding affinity to targeted pathogens, thus enhancing the therapeutic arsenal against bacterial infections [204]. In a different but highly innovative approach, a research team at Wageningen University (The Netherlands) initiated the Xylencer project, a bacteriophage therapy targeting *Xff* that utilises genetically engineered phages with enhanced binding to both their insect vectors and target bacteria [33] (see in Supplementary Material S2).

### 5.2. Recombinant Phage Enzymes: A Safe Way to Bypass Dubious Phage Genomes

Recombinant phage-derived enzymes offer a promising and safe alternative to whole-phage therapy, circumventing concerns associated with temperate or poorly characterised phage genomes [205,206,207,208]. Phages encode numerous lytic enzymes, such as capsule depolymerases, virion-associated lysins (VALs), and endolysins, that naturally degrade bacterial surface structures during different stages of the infection cycle (Figure 1) [209,210]. When produced recombinantly, these enzymes can be characterised, standardised, and applied with far greater predictability than intact virions. Compared with endolysins, VALs share several advantages: like depolymerases, being capsid-associated structural proteins, they generally exhibit greater stability and environmental tolerance [211]. Importantly, resistance against lytic enzymes appears rare, as endolysins target highly conserved peptidoglycan, while depolymerases act non-lytically on polysaccharide capsules without selecting for resistance [189,207,208,209,212]. Recombinant lysins have demonstrated strong activity against Gram-positive pathogens, including MRSA [213,214,215,216], and depolymerases show considerable promise against encapsulated Gram-negative strains such as *Klebsiella pneumoniae* [207,217,218,219,220]. Combining lysins that act on different cleavage sites often results in synergistic effects, and lysins can also be used in combination with antibiotics [221]. Their modular architecture also enables engineering of enhanced variants through domain shuffling, truncation of nonessential regions, or construction of synergistic chimeric enzymes, further expanding their utility [219,222]. Although not the central focus of whole-phage biocontrol, recombinant phage enzymes represent an increasingly powerful complementary strategy, with growing relevance in the era of antibiotic resistance [211,213,215,218,223].

### 5.3. AI-Driven Discovery and Optimisation

The complexity of optimising advanced phage preparations, which involves balancing synergy, host range, and resistance mitigation, has transitioned from empirical trial-and-error to predictive design through artificial intelligence (AI) and machine learning (ML). Tools like PhageAI can analyse phage nucleotide sequences to accurately classify phages (e.g., virulent versus temperate) and predict life cycles and bacterial resistance pathways, providing a necessary layer of safety and specificity and ensuring the long-term efficacy of phage therapy. It simultaneously represents a repository of knowledge of bacteriophages and a bioinformatics pipeline to analyse genomes with artificial intelligence support [224].

More recently, AI has moved into the realm of generative design. King and colleagues [38] used frontier genome language models, Evo 1 and Evo 2, to design the first artificially generated, viable bacteriophage genomes, creating phages with enhanced fitness and novelty compared with natural templates. They generated whole bacteriophage genomes, using the lytic phage ΦX174 of *Escherichia coli* as a design template. Experimental testing of these AI-generated genomes produced 16 viable phages with substantial evolutionary novelty. Cryo-electron microscopy revealed that one of the phages incorporates an evolutionarily distant DNA packaging protein into its capsid. Several of the generated phages exhibited greater fitness than ΦX174, both in growth competitions and in lysis kinetics. A cocktail composed of these phages rapidly overcame ΦX174 resistance in three *E. coli* strains, highlighting the potential of AI-designed phages for therapeutic applications against rapidly evolving bacterial pathogens. This work lays a foundation for the generative design of diverse synthetic phages and, more broadly, functional living systems at the genome scale [38]. This AI-driven approach, alongside ML-based pipelines for predicting phage–host interactions [225,226], is crucial for decoding the infinite potential combinations of phage–bacterial genomic interactions, enabling phage therapy to scale efficiently against rapidly evolving pathogens.

## 6. Conclusions and Future Prospects

Several valuable review articles and book chapters were presented in the past few years about the topic of phage therapy in plant protection, each focusing on different aspects and details of phage therapy, such as phage ecology, historical roots, challenges, and future prospects [85,106,168,227,228]. The century-long history of phage therapy in plant disease management illustrates not a linear technological progression, but a recurring pattern of promise, neglect, and rediscovery shaped by broader agricultural paradigms. While early enthusiasm was eclipsed by the antibiotic era, the contemporary resurgence of phage-based biocontrol has been driven less by novelty than by necessity: specifically, by the failure of chemical bactericides to remain effective, sustainable, or socially acceptable. Today, phage therapy is no longer positioned merely as a “natural alternative,” but as a highly knowledge-intensive, precision-driven intervention whose success depends on biological, technological, and regulatory alignment.

Despite the substantial body of experimental evidence summarised in this review, several unresolved issues continue to limit the routine deployment of phage therapy in agriculture.

First, predictability and robustness under field conditions remain insufficiently understood. Phage efficacy is often demonstrated in controlled greenhouse or short-term field trials, yet long-term performance across seasons, climates, and cropping systems is poorly characterised [229]. A major contributing factor is the absence of systematic dose–response relationships, particularly when comparing soil drench and foliar spray applications. In the case of spraying, the dose–response curve is unstable, decreasing rapidly over time due to various environmental effects on phages, including UV inactivation. Therefore, successful control requires a higher initial dose, use of protective formulations, and/or application during the evening. In the case of soil irrigation, the curve is more stable. However, the adsorption capacity of the soil can significantly shift the response threshold required to achieve successful control. The development of the dose–response curve may be affected in the long term by the development of bacterial resistance to bacteriophage infection. Another problem is that phage concentrations, timing, and frequency of application vary widely between studies, limiting both rational optimisation and comparability between studies. Establishing standardised, crop- and application-specific dose–response frameworks is therefore a critical and tractable research priority.

Second, the evolutionary dynamics between phages, bacterial pathogens, and plant-associated microbiomes remain an open question. While phage resistance frequently incurs fitness costs, the durability of these trade-offs under agricultural selection pressure is uncertain. Importantly, resistance emergence has rarely been examined under realistic agronomic conditions, such as mixed cropping systems, spatial heterogeneity, or repeated seasonal applications. The lack of predictive resistance insurgence models means that resistance is still treated largely as a qualitative concern rather than a quantifiable risk. Integrating evolutionary modelling with field-scale experiments will be essential to assess resistance trajectories over multiple growing cycles and to inform rational phage cocktail design.

Third, regulatory incompatibility with the biological nature of phages represents a structural bottleneck. Existing pesticide authorisation frameworks (particularly in the European Union) are poorly suited to agents that are inherently adaptive, strain-specific, and periodically reformulated. The requirement to repeatedly register modified phage cocktails undermines one of their key advantages: responsiveness to emerging resistance. Without regulatory models that accommodate controlled adaptability, phage therapy risks remain confined to temporary authorisations or niche applications.

Fourth, economic feasibility remains insufficiently quantified. Although numerous studies report disease suppression comparable with copper-based treatments, formal cost–benefit analyses of phage applications versus standard copper sprays are largely absent. Without rigorous comparisons that include formulation and production costs, application frequency, yield protection, and environmental externalities, the practical competitiveness of phage-based products cannot be reliably assessed. This gap limits evidence-based decision-making by growers and regulators alike.

**Figure 3:**
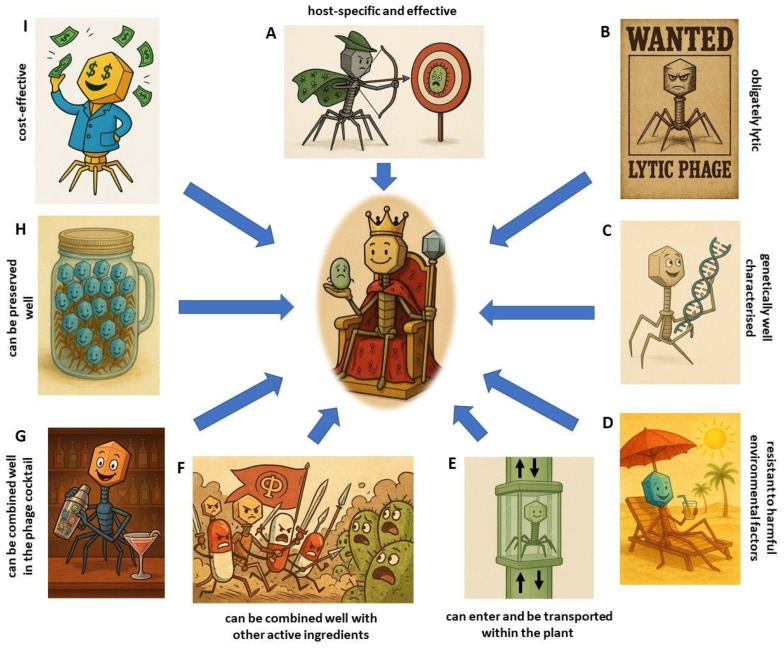
The perfect phage (PP). The perfect bacteriophage suitable for use in plant protection has all or most of the following properties: (**A**) host-specific and can kill the target bacteria with high efficiency; (**B**) they are obligately lytic (virulent phages), meaning that they kill bacteria instead of integrating into the host genome (i.e., they are incapable of transduction); (**C**) they are genetically well characterised and certainly do not carry virulence or toxin genes; (**D**) they are resistant to the destructive effects of the environment (UV light, temperature, pH, dehydration, etc.); (**E**) they can enter the plant through the leaves and/or roots and be transported within the plant (The black arrows indicate the up- and down-stream transport of phages into the plant); (**F**) they can be combined well with other active ingredients that either act directly on the bacteria or through the plant defence system; (**G**) they can be combined well in the phage cocktail by complementing each other’s host specificity and maintaining high individual effectiveness, which helps reduce the risk of phage resistance development; (**H**) phages can be stored with little loss of effectiveness from production to use; (**I**) their production, handling, and application during plant protection are economical. The blue arrows indicate that the perfect phage in the middle has all the favorable properties. (The pictures were generated with ChatGPT-4.1 with special prompts).

The concept of the “perfect phage” (Figure 3) provides a useful theoretical benchmark rather than a realistic endpoint. No naturally occurring or engineered phage is likely to satisfy all listed criteria simultaneously. Instead, the figure highlights inherent trade-offs between host specificity and robustness, between evolutionary flexibility and regulatory stability, and between biological efficacy and economic feasibility. Recognising these trade-offs is essential for rational product design and realistic expectation setting.

Looking forward, the future of phage therapy in plant protection will depend less on single technological breakthroughs than on systems-level integration. Synthetic biology, AI-assisted phage design, advanced formulation technologies, and microbiome-aware application strategies offer important tools, but they cannot compensate for weak regulatory alignment or missing agronomic data. Phage-based products are most likely to succeed as components of integrated disease management strategies, combined with resistant cultivars, beneficial microbes, plant defence inducers, and reduced chemical inputs.

In conclusion, as the world faces a post-antibiotic era and an urgent need for sustainable, precision-based disease control, bacteriophages, the oldest antibacterial agents of nature, are poised to become vital tools once again. However, the widespread adoption will require confronting unresolved biological uncertainties, regulatory inertia, and economic constraints directly, while fostering interdisciplinary collaboration and integrating phage-based strategies into the broader One Health framework that links human, animal, and environmental health. The next phase of phage therapy research must therefore shift from demonstrating that phages “can work” toward defining “how much”, “how often”, “where”, and “at what cost” they work reliably.

## Figures and Tables

**Table 2:** Phage products in plant protection (USA, EU).

Product Name	Company Credited	Registering Authority/Registration Details (Year)	Target Species (Diseases)
AgriPhage	Omnilytics Inc. (Sandy, UT, USA)	EPA US/Reg. 67986-1 (2005)	*Xanthomonas* spp.; *Pseudomonas syringae* pv. *tomato* (bacterial spot and speck on tomato/pepper)
AgriPhage—Tomato Canker	Omnilytics Inc. (Sandy, UT, USA)	EPA US/Reg. 67986-6 (2011)	*Clavibacter michiganensis* subsp. *michiganensis* (tomato bacterial canker)
AgriPhage—Fire Blight	Omnilytics Inc. (Sandy, UT, USA)	EPA US/Reg. 67986-8 (2020)	*Erwinia amylovora* (fire blight)
AgriPhage—Citrus Canker	Omnilytics Inc. (Sandy, UT, USA)	EPA US/Reg. 67986-9 (2018)	*Xanthomonas citri* pv. *citri* (citrus bacterial canker)
AgriPhage—Nut & Stone Fruit	Omnilytics Inc. (Sandy, UT, USA)	EPA US/Reg. 67986-10 (2023)	*Xanthomonas arboricola* pv. *pruni*, *X. arboricola* pv. *juglandis*, *X. arboricola* pv. *corylina*; *Pseudomonas syringae* pv. *syringae* (diseases of stone fruits/nuts)
AgriPhage CMM	Omnilytics Inc., (Sandy, UT, USA)	PMRA Canada/RD2012–21/ (2012)	*Clavibacter michiganensis* subsp. *michiganensis* (bacterial canker of tomatoes)
ERWIPHAGE PLUS	Enviroinvest Corp. (Pécs, Hungary)	Hungary (Temporary authorisation for Spring 2018)	*Erwinia amylovora* (fire blight)
XylPhi-PD	Otsuka Pharmaceutical Co. Ltd. (Tokyo, Japan); A&P Inphatec (Palo Alto, CA, USA)	EPA US/Reg 92918-1 (2021)	*Xylella fastidiosa* subsp. *fastidiosa* (Pierce’s disease)

## Data Availability

Data are contained within the article and Supplementary Materials.

## Supplementary Materials

The following supporting information can be downloaded at: https://www.mdpi.com/article/10.3390/plants15030368/s1, S1: Literature Sources and Selection Strategy; S2: Bacteriophage-Based Biocontrol, A Detailed Review of Milestone Studies; S3: Overview of the *in vivo* efficacy of bacteriophages in different pathosystems.
