# Supplementary material for: Phage Therapy in Plant Disease Management: 110 Years of History, Current Challenges, and Future Trends
Source: Plants (Basel). 2026 Jan 24;15(3):368. doi: 10.3390/plants15030368 (PMC12899248; doi:10.3390/plants15030368)
Supplement: Supplementary file 1 [file plants-15-00368-s001.zip › Supplementary Material S1. Literature Sources and Selection Strategy.pdf]

plants

MDPI

Supplementary material

Phage Therapy in Plant Disease Management: 110 Years of History, Current Challenges, and Future Trends

Botond Zsombor Pertics, Lóránt Király, Zoltán Bozsó *, Dániel Krüzselyi, Judit Kolozsváriné Nagy, András Künstler, Ferenc Samu and Ildikó Schwarczinger *

Plant Protection Institute, Hungarian Research Network Centre for Agricultural Research, Fehérvári út 132–144, 1116 Budapest, Hungary; pertics.botond@atk.hun-ren.hu (B.Z.P.); kiraly.lorant@atk.hun-ren.hu (L.K.); kruzselyi.daniel@atk.hun-ren.hu (D.K.); nagy.judit@atk.hun-ren.hu (J.K.N.); kunstler.andras@atk.hun-ren.hu (A.K.); samu.ferenc@atk.hun-ren.hu (F.S.)

* Correspondence: bozso.zoltan@atk.hun-ren.hu (Z.B.); schwarczinger.ildiko@atk.hun-ren.hu (I.S.); Tel.: +36-30-486-2416 (Z.B.)

Literature Sources and Selection Strategy

This article is a narrative and historical review aiming to synthesize key scientific, clinical, and agricultural milestones in the development of bacteriophage research and phage therapy. The literature was identified through targeted searches in major scientific databases, including Web of Science, Scopus, PubMed, and Google Scholar, JSTOR covering publications from 1915 to 2025. Boolean search string were used in various combinations and included: "bacteriophage", "phage therapy", "plant disease", "plant pathogen", "phytopathogenic bacteria", "phage biocontrol", "agricultural phages" . Given the historical scope of the review, grey literature sources were also consulted, including historically relevant book chapters, early experimental reports, conference proceedings, patents, and institutional or regulatory documents related to phage applications in agriculture.

Studies were included if they reported experimental, applied, or conceptual advances related to bacteriophages or phage therapy in plant disease management, including laboratory, greenhouse, field, and commercial phage-based products. Studies focusing exclusively on human or veterinary applications without relevance to agricultural or plant disease management contexts were excluded from the present review, except which represented major milestones in the historical or conceptual development of phage therapy. Due to the broad time span, heterogeneity of study designs, and the conceptual nature of the review, a formal PRISMA-based systematic workflow was not applied. Nevertheless, the literature selection followed principles of transparency and reproducibility appropriate for narrative historical reviews.
