# Supplementary material for: Phage Therapy in Plant Disease Management: 110 Years of History, Current Challenges, and Future Trends
Source: Plants (Basel). 2026 Jan 24;15(3):368. doi: 10.3390/plants15030368 (PMC12899248; doi:10.3390/plants15030368)
Supplement: Supplementary file 1 [file plants-15-00368-s001.zip › Supplementary Material S2. Bacteriophage-Based Biocontrol, A Detailed Review of Milestone Studies.pdf]

plants

MDPI

Supplementary material

Phage Therapy in Plant Disease Management: 110 Years of History, Current Challenges, and Future Trends

Botond Zsombor Pertics, Lóránt Király, Zoltán Bozsó *, Dániel Krüzselyi, Judit Kolozsváriné Nagy, András Künstler, Ferenc Samu and Ildikó Schwarczinger *

Plant Protection Institute, Hungarian Research Network Centre for Agricultural Research, Fehérvári út 132–144, 1116 Budapest, Hungary; pertics.botond@atk.hun-ren.hu (B.Z.P.); kiraly.lorant@atk.hun-ren.hu (L.K.); kruzselyi.daniel@atk.hun-ren.hu (D.K.); nagy.judit@atk.hun-ren.hu (J.K.N.); kunstler.andras@atk.hun-ren.hu (A.K.); samu.ferenc@atk.hun-ren.hu (F.S.)

* Correspondence: bozso.zoltan@atk.hun-ren.hu (Z.B.); schwarczinger.ildiko@atk.hun-ren.hu (I.S.); Tel.: +36-30-486-2416 (Z.B.)

1. Bacteriophage-Based Biocontrol Strategies Against Major Crop Pathogens: A Detailed Review of Milestone Studies (2000-2025)

1.1. Phage Control of Potato Diseases; Soft Rot and Blackleg Caused by *Pectobacterium* Species and *Dickeya solani*, Bacterial Wilt Caused by *Ralstonia solanacearum* and Common Scab, Caused by *Streptomyces scabies*

Experiments with *Myoviridae* phages (LIMEstone1, LIMEstone2) isolated from soil samples of a potato field in Belgium by Adriaenssens et al. [1] revealed that potato (cvs. Bintje and Kondor) tubers inoculated with *D. solani* alone developed up to 40% of tissue maceration, while tubers co-inoculated with bacteria+phage had only 10% maceration. Field trials with the same phages and pathogen demonstrated that the biocontrol effect was also effective *in planta*, as phage treatments (10^{10} plaque forming unit (PFU) per ml) of *D. solani*-infected potato seed tubers led to a 13% increase in yield and 5% reduction in disease incidence. Similar research by a Polish group demonstrated that ϕD phages (*Myoviridae*) derived from soil samples of different regions of Poland could prevent tissue maceration in bacteria-infected potato tubers by up to 70% [2]. Regarding soft rot disease caused by *Pectobacterium* species, a combined or individual application of *Myoviridae* phages (ϕPD10.3, ϕPD23.1) at 10^5 PFU/ml prevented potato tissue maceration by 80% in potato slices and by 95% in whole tubers following a mixed infection by *P. carotovorum* subsp. *carotovorum* (*Pcc*) and *P. wasabie* [3]. Importantly, phages isolated from soil and water samples displayed cross reactivity not only against *P. atrosepticum* but also towards two phage-resistant *P. carotovorum* strains [4]. It is worth mentioning that soft rot infections caused by *Pcc* have been also successfully controlled by phage applications in other vegetable crops like lettuce, onion and Chinese cabbage [5–7].

Remarkably, a cocktail of different phages reduced incidence of potato soft rot caused by *D. solani* from 93% to 49%, and disease severity by 75% following five days of storage after bacterial infection [8]. Furthermore, phages targeting *P. atrosepticum* could prevent maceration of harvested potatoes [9]. Phages of *P. carotovorum* and *D. solani* conferred a reduction of up to 80% in soft rot severity during storage of fruits and vegatables [10]. The above examples point to the potential of phage therapy beyond field/greenhouse applications: the control of storage diseases caused by pectolytic bacteria (for two recent reviews see [11,12]. In fact, phage applications may decrease pesticide dependence during fruit and vegetable storage and transport, a particularly relevant aspect for crops with strict residue limits.

Brown rot (bacterial wilt) of the potato plant is caused by one of the most destructive phytopathogenic soil-borne bacterium, *R. solanacearum*. A phage cocktail (10^8 PFU/ml) was formulated from *Siphoviridae* phages, isolated from water samples, that could efficiently lyse *R. solanacearum* cells. Furthermore, this phage cocktail provided a high degree (80%) protection from symptoms in preventive treatments and a delayed disease development in curative applications. The phages could also reduce bacterial density in contaminated soils [13]. In a different study, no symptoms of bacterial wilt were observed in phage treated, *R. solanacearum*-infected potatoes and qPCR assays indeed revealed significantly reduced bacterial titres in the soil [14].

Another economically important potato disease caused by the Gram-positive bacterium *Streptomyces scabies* is common scab (formation of corky lesions on tubers). It was shown that treatments with the phage ϕAS1 (*Siphoviridae*) is highly efficient in potato seed tubers, since it resulted in a tuber progeny with only 1.2% scab symptoms as compared to 23% in non-treated controls [15]. Common scab, however, is also a problem in other vegetable crops. In radish seedlings the application of either of two *Siphoviridae* phages (Stsc1 and Stsc3) could prevent the development of common scab symptoms. Furthermore, phages Stsc1 and Stsc3 could infect 88% and 75% of the tested pathogenic *S. scabies* strains, respectively [16].

1.2. Phage Control of Tomato, Pepper and Crucifer Diseases Caused by *R. solanacearum* and *X. campestris* Pathovars

Bacterial wilt, caused by *R. solanacearum*, is also one of the most important bacterial diseases of cultivated tomatoes. However, tomato plants treated with the phage ϕRSL1 (*Myoviridae*) did not display any wilting symptoms, while all untreated plants had wilted by 18 days after infection. Importantly, active (infective) ϕRSL1 particles could be recovered from roots of treated plants and also from soils 4 months after infection [17]. In a similar study, the simultaneous application of a *Podoviridae* phage (PE204) and *R. solanacearum* in the rhizosphere of tomato completely inhibited the occurrence of bacterial wilt. Furthermore, the addition of Silwet L-77 to the phage suspension did not impair disease control activity, suggesting the perspective of potentially successful field applications [18]. Other groups reported that phage (cocktail) treatments (10^6 and 10^{11} PFU/ml) of tomato plants suppressed bacterial wilt up to 80-100%, even under field conditions [14,19], while the co-inoculation of *R. solanacearum* and *Podoviridae* phages (10^6-10^{10} PFU/ml) by irrigation significantly decreased wilt incidence in biocontrol assays by 20–80% [20]. Although soil drenching of *R. solanacearum*-infected tomato seedlings with a single *Myoviridae* phage resulted only in a partial suppression of wilt symptoms, bacterial titers were significantly (87%) reduced [21]. Importantly, recent research using a *Podoviridae* cocktail (10^5 PFU/ml) showed that repeated applications increased the *R. solanacearum* suppression efficacy from ~33-40% to 67-84% [22].

Bacterial spot of tomato and also pepper is caused by *Xathomonas campestris* pv. *vesicatoria (Xcv)*. One of the first reports on the successful phage-based control of this disease in tomato demonstrated that a phage cocktail including a mixture of host-range mutant (h-mutant) bacteriophages reduces the severity of bacterial spot by 14% in field experiments, with an even better protective effect (40% symptom reduction) under greenhouse conditions [23]. Subsequent greenhouse and field experiments with tomato involved the testing of formulated phage cocktails against bacterial spot and revealed that protective formulations provided a better control of *Xcv* than unformulated phages [24]. In another series of field experiments, a formulation of six different phages specific to *Xcv* race T3 strain 91–118 was used. Phage treatments gave an effect comparable to the standard disease control protocol using copper-mancozeb [25].

Another *Xathomonas* species, *X. campestris* pv. *campestris* (*Xcc*) causes the black rot disease in crucifer crops. In field experiments, a combination of a phage solution (10^7

PFU/ml) with a non-pathogenic *Xcc* strain resulted in a disease incidence in broccoli that was 59% of that in non-pre-treated controls. It was also shown that the non-pathogenic *Xcc* strain, when used together with the phage, was 10% more effective than the phage alone [26]. According to Holtappels et al. [27], preventive foliar spraying with phage solution inhibits black rot better than phage + bacteria co-inoculation. Under field conditions, a preventive application of a cocktail of two phages led to a 30% reduction in numbers of symptomatic plants, as compared to non-treated controls [27]. In addition, a very recent work demonstrated that application of a single phage formulation on kohlrabi leaves resulted in significantly reduced *Xcc* disease symptoms (late or no necrosis on leaves) [28].

1.3. Phage Control of Fruit Crop Diseases Caused by *P. syringae* Pathovars, *E. amylovora* and *X. fastidiosa* subsp. *fastidiosa*

Pseudomonas syringae pathovars cause bacterial canker and blight diseases of different fruit crops. For example, isolation of *Myoviridae* and *Podoviridae* phages from soil samples in South Korean kiwifruit orchards revealed their potential in controlling canker of this globally important fruit crop. The phages were active against *P. syringae* pv. *actinidiae* (*Psa*) strains (e.g. Psa2 and Psa3), furthermore, some of them could also inhibit other *P. syringae* pathovars [29]. A Chilean group reported that mixtures of *Podoviridae* phages (10^8 PFU/ml) could reduce bacterial titres on kiwi leaves 24 hours after *Psa* infection by at least 75% as compared to untreated plants [30]. Also, a *Cystoviridae* phage (ϕ6) proved to be effective against two biovar 3 *Psa* strains highly pathogenic on kiwi both *in vitro* and *ex vivo*. Importantly, one of the inactivated *Psa* strains (CRA-FRU 14.10) could not re-grow when the phage treatments were finished [31]). Combining *Myoviridae* phages (PN05, PN09) with carvacrol suppressed *Psa* concentrations, biofilm development, and resistance emergence [32].

Bacterial canker of *Prunus* species including cherry is caused by *P. syringae* pv. *syringae* and pv. *morsprunorum*. Two different cocktails of several *Podoviridae* and *Myoviridae* phages (10^8 PFU/ml) could significantly suppress bacterial populations both on cherry leaves and twigs. A curative treatment of cherry seedlings and wounded cherry twigs one day after bacterial inoculation by phage sprayings controlled bacterial titers for several weeks [33]. It is worth mentioning that *P. syringae* pv. *porri* can cause bacterial blight of a vegetable, i. e. leek. Although application of a cocktail of *Myoviridae* phages (10^7-10^8 PFU/ml) significantly attenuated symptoms on leek even in a field experiment, it could not completely stop the infection, likely due to development of bacterial mutations in the LPS capsule that resulted in phage-resistance [34].

Fire blight is one of the most destructive diseases of fruit trees like apple and pear, caused by the bacterium *Erwinia amylovora*. Using the epiphytic bacterium *Pantoea agglomerans* Eh21-5 as a carrier, phages (ϕEa1337-26 (*Podoviridae*) and ϕEa2345-6 (*Myoviridae*)) reduced infection on detached pear blossoms by 84% and 96%, respectively. In addition, the phage ϕEa2345-6 (with the Eh21-5 carrier) suppressed *E. amylovora* infection on flowers of potted apple trees with an effect comparable to that of the antibiotic streptomycin [35]. In our own study, we isolated several bacteriophages from blighted apple and pear trees from different areas of Hungary. A combination of three selected phage isolates (ΦEaH2A, ΦEaH5K and ΦEaH7B) significantly reduced both bacterial multiplication and fire blight symptoms in apple blossoms and green pear fruit slices. Specifically, open flowers receiving a phage cocktail on their pistils before bacterial infection displayed a 65-84 % reduction of *E. amylovora* multiplication. Furthermore, pear slices soaked in the phage cocktail and dried, before bacterial inoculation, also showed a significant reduction in symptom severity [36]. Importantly, it was demonstrated that stem injections of of one-year old pear plants with single phages have also prevented fire blight symptoms similarly to antibiotics [37]. An innovative approach by Born and co-workers (2017) involved the engineering

of phage Y2 by an EPS depolymerase, in order to improve efficiency of bacterial cell lysis. Detached apple flowers were sprayed with the phage (3 x 10^8 PFU/ml) one hour after inoculation by *E. amylovora*, resulting in a reduction of bacterial titers up to 81-95% [38]. In two recent studies, preventive applications of single phages on detached fruits (loquat, pear), resulted a significant reduction (66-100%) in disease severity (smaller fire blight lesions) [28,39].

Xylella fastidiosa subsp. *fastidiosa* (*Xff*) is considered as one of the most dangerous plant-pathogenic bacteria worldwide, infecting several fruit crops (e.g. citrus, almond, olives, peach). In grapevine, *Xff* causes Pierce's disease (PD), with leaf chlorosis, scorching and wilting, as the bacteria block xylem vessels, often causing host plant death. In order to control PD and its causal agent, a cocktail with four lytic phages was developed. Preventive and curative treatments with the phage cocktail solution (10^{10} PFU/ml) not only prevented PD symptoms but also significantly (10 to 1000-fold) reduced bacterial numbers. The efficacy and possible potential of this phage application treatment is also indicated by the fact that no phage-resistant *Xff* isolates were recovered *in planta* [40].

In a different but highly innovative approach, a research team at Wageningen University (The Netherlands) initiated the Xylencer project, a bacteriophage therapy for *Xff* that utilizes genetically engineered phages. Efficient phage delivery is ensured by inserting the phage genome into a plasmid within the phage delivery bacterium (a *X. arboricola* isolate) injected as a solution into grapevine trunks. Xylencer phages encode for a Pathogen Associated Molecular Pattern (PAMP) so that phage infection and bacterial lysis may also trigger plant immune responses. In addition, Xylencer phages bind to the mouthparts of vector insects through their capsid, thus allowing a self-spreading mechanism of phages to reach all bacteria-infected plants (XYLENCER project) [41].

References

1. Adriaenssens, E.M.; van Vaerenbergh, J.; Vandenheuvel, D.; Dunon, V.; Ceyssens, P.J.; de Proft, M.; Kropinski, A.M.; Noben, J.P.; Maes, M.; Lavigne, R. T4-Related Bacteriophage limestone Isolates for the Control of Soft Rot on Potato Caused by "*Dickeya solani*." *PLoS One* **2012**, *7*, e33227, doi:10.1371/journal.pone.0033227.
2. Czajkowski, R.; Ozymko, Z.; Zwirowski, S.; Lojkowska, E. Complete Genome Sequence of a Broad-Host-Range Lytic *Dickeya* spp. Bacteriophage ΦD5. *Arch. Virol.* **2014**, *159*, 3153–3155, doi:10.1007/s00705-014-2170-8.
3. Czajkowski, R.; Ozymko, Z.; De Jager, V.; Siwinska, J.; Smolarska, A.; Ossowicki, A.; Narajczyk, M.; Lojkowska, E. Genomic, Proteomic and Morphological Characterization of Two Novel Broad Host Lytic Bacteriophages ΦPD10.3 and ΦPD23.1 Infecting Pectinolytic *Pectobacterium* spp. and *Dickeya* spp. *PLoS One* **2015**, *10*, e0119812, doi:10.1371/JOURNAL.PONE.0119812.
4. Muturi, P.; Yu, J.; Maina, A.N.; Kariuki, S.; Mwaura, F.B.; Wei, H. Bacteriophages Isolated in China for the Control of *Pectobacterium carotovorum* Causing Potato Soft Rot in Kenya. *Virol. Sin.* **2019**, *34*, 287–294, doi:10.1007/s12250-019-00091-7.
5. Lim, J.A.; Jee, S.; Lee, D.H.; Roh, E.; Jung, K.; Oh, C.; Heu, S. Biocontrol of *Pectobacterium carotovorum* subsp. *carotovorum* Using Bacteriophage PP1. *J. Microbiol. Biotechnol.* **2013**, *23*, 1147–1153, doi:10.4014/jmb.1304.04001.
6. Zaczek-Moczydłowska, M.A.; Young, G.K.; Trudgett, J.; Fleming, C.C.; Campbell, K.; O'Hanlon, R. Genomic Characterization, Formulation and Efficacy in Planta of a Siphoviridae and Podoviridae Protection Cocktail against the Bacterial Plant Pathogens Pectobacterium Spp. *Viruses* **2020**, *12*, 150, doi:10.3390/v12020150.
7. Vu, N.T.; Kim, H.; Lee, S.; Hwang, I.S.; Kwon, C.T.; Oh, C.S. Bacteriophage Cocktail for Biocontrol of Soft Rot Disease Caused by *Pectobacterium* Species in Chinese Cabbage. *Appl. Microbiol. Biotechnol.* **2024**, *108*, 1–15, doi:10.1007/s00253-023-12881-x.
8. Carstens, A.B.; Djurhuus, A.M.; Kot, W.; Jacobs-Sera, D.; Hatfull, G.F.; Hansen, L.H. Unlocking the Potential of 46 New Bacteriophages for Biocontrol of Dickeya Solani. *Viruses* **2018**, *10*, 621, doi:10.3390/v10110621.
9. Kmoch, M.; Vacek, J.; Loubová, V.; Petrzik, K.; Brázdová, S.; Ševčík, R. Potential of Limestonevirus Bacteriophages for Ecological Control of Dickeya Solani Causing Bacterial Potato Blackleg. *Agric.* **2024**, *14*, 497, doi:10.3390/agriculture14030497.
10. Zaczek, M.; Weber-Dabrowska, B.; Górski, A. Phages in the Global Fruit and Vegetable Industry. *J. Appl. Microbiol.* **2015**, *118*,

537–556, doi:10.1111/jam.12700.

11. Hoffmann, A.; Sadowska, K.; Zenelt, W.; Krawczyk, K. Bacteriophages as a Sustainable Tool for Plant Disease Management: Benefits and Challenges. *Agronomy* **2025**, *15*, 2507, doi:10.3390/agronomy15112507.
12. Hoffmann, A.; Sadowska, K.; Zenelt, W.; Krawczyk, K. Post-Harvest Disease Control Using Bacteriophages: Current Strategies, Practical Applications, and Future Trends. *Agric.* **2025**, *15*, 2261, doi:10.3390/agriculture15212261.
13. Wei, C.; Liu, J.; Maina, A.N.; Mwaura, F.B.; Yu, J.; Yan, C.; Zhang, R.; Wei, H. Developing a Bacteriophage Cocktail for Biocontrol of Potato Bacterial Wilt. *Virol. Sin.* **2017**, *32*, 476–484, doi:10.1007/s12250-017-3987-6.
14. Elhalag, K.; Eldin, M.N.; Hussien, A.; Ahmad, A. Potential Use of Soilborne Lytic Podoviridae Phage as a Biocontrol Agent against *Ralstonia solanacearum*. *J. Basic Microbiol.* **2018**, *58*, 658–669, doi:10.1002/jobm.201800039.
15. McKenna, F.; El-Tarabily, K.A.; Hardy, G.E.S.T.J.; Dell, B. Novel in Vivo Use of a Polyvalent *Streptomyces* Phage to Disinfest *Streptomyces scabies*-Infected Seed Potatoes. *Plant Pathol.* **2001**, *50*, 666–675, doi:10.1046/j.1365-3059.2001.00648.x.
16. Goyer, C. Isolation and Characterization of Phages Stsc1 and Stsc3 Infecting *Streptomyces scabies* and Their Potential as Biocontrol Agents. *Can. J. Plant Pathol.* **2005**, *27*, 210–216, doi:10.1080/07060660509507218.
17. Fujiwara, A.; Fujisawa, M.; Hamasaki, R.; Kawasaki, T.; Fujie, M.; Yamada, T. Biocontrol of *Ralstonia solanacearum* by Treatment with Lytic Bacteriophages. *Appl. Environ. Microbiol.* **2011**, *77*, 4155–4162, doi:10.1128/AEM.02847-10.
18. Bae, Y.J.; Wu, J.; Lee, H.J.; Jo, E.J.; Murugaiyan, S.; Chung, E.; Lee, S.W. Biocontrol Potential of a Lytic Bacteriophage PE204 against Bacterial Wilt of Tomato. *J. Microbiol. Biotechnol.* **2012**, *22*, 1613–1620, doi:10.4014/jmb.1208.08072.
19. Wang, X.; Wei, Z.; Yang, K.; Wang, J.; Jousset, A.; Xu, Y.; Shen, Q.; Friman, V.P. Phage Combination Therapies for Bacterial Wilt Disease in Tomato. *Nat. Biotechnol.* **2019**, *37*, 1513–1520, doi:10.1038/s41587-019-0328-3.
20. Álvarez, B.; López, M.M.; Biosca, E.G. Biocontrol of the Major Plant Pathogen *Ralstonia solanacearum* in Irrigation Water and Host Plants by Novel Waterborne Lytic Bacteriophages. *Front. Microbiol.* **2019**, *10*, 492073, doi:10.3389/fmicb.2019.02813.
21. Umrao, P.D.; Kumar, V.; Kaistha, S.D. Biocontrol Potential of Bacteriophage ɸsp1 against Bacterial Wilt-Causing *Ralstonia solanacearum* in Solanaceae Crops. *Egypt. J. Biol. Pest Control* **2021**, *31*, 61-, doi:10.1186/s41938-021-00408-3.
22. Wang, X.; Wang, S.; Huang, M.; He, Y.; Guo, S.; Yang, K.; Wang, N.; Sun, T.; Yang, H.; Yang, T.; et al. Phages Enhance Both Phytopathogen Density Control and Rhizosphere Microbiome Suppressiveness. *MBio* **2024**, *15*, doi:10.1128/mbio.03016-23.
23. Flaherty, J.E.; Jones, J.B.; Harbaugh, B.K.; Somodi, G.C.; Jackson, L.E. Control of Bacterial Spot on Tomato in the Greenhouse and Field with H-Mutant Bacteriophages. *HortScience* **2000**, *35*, 882–884, doi:10.21273/hortsci.35.5.882.
24. Balogh, B.; Jones, J.B.; Momol, M.T.; Olson, S.M.; Obradovic, A.; King, P.; Jackson, L.E. Improved Efficacy of Newly Formulated Bacteriophages for Management of Bacterial Spot on Tomato. *Plant Dis.* **2003**, *87*, 949–954, doi:10.1094/PDIS.2003.87.8.949.
25. Obradovic, A.; Jones, J.B.; Momol, M.T.; Balogh, B.; Olson, S.M. Management of Tomato Bacterial Spot in the Field by Foliar Applications of Bacteriophages and SAR Inducers. *Plant Dis.* **2004**, *88*, 736–740, doi:10.1094/PDIS.2004.88.7.736.
26. Nagai, H.; Miyake, N.; Kato, S.; Maekawa, D.; Inoue, Y.; Takikawa, Y. Improved Control of Black Rot of Broccoli Caused by *Xanthomonas campestris* pv. *campestris* Using a Bacteriophage and a Nonpathogenic Xanthomonas Sp. Strain. *J. Gen. Plant Pathol.* **2017**, *83*, 373–381, doi:10.1007/s10327-017-0745-4.
27. Holtappels, D.; Fortuna, K.J.; Moons, L.; Broeckaert, N.; Bäcker, L.E.; Venneman, S.; Rombouts, S.; Lippens, L.; Baeyen, S.; Pollet, S.; et al. The Potential of Bacteriophages to Control *Xanthomonas campestris* pv. *campestris* at Different Stages of Disease Development. *Microb. Biotechnol.* **2022**, *15*, 1762–1782, doi:10.1111/1751-7915.14004.
28. Vique, G.; Mendoza-Barberá, E.; Ramos-Barbero, M.D.; Blanco-Picazo, P.; Sala-Comorera, L.; Quirós, P.; Atares, S.; Salaet, I.; Muniesa, M.; Rodríguez-Rubio, L. Efficacy of *Erwinia amylovora* and *Xanthomonas campestris* pv *campestris* Phages to Control Fire Blight and Black Rot in Vivo . *Microbiol. Spectr.* **2025**, *13*, doi:10.1128/spectrum.00280-25.
29. Yu, J.G.; Lim, J.A.; Song, Y.R.; Heu, S.; Kim, G.H.; Koh, Y.J.; Oh, C.S. Isolation and Characterization of Bacteriophages against *Pseudomonas syringae* pv. *actinidiae* Causing Bacterial Canker Disease in Kiwifruit. *J. Microbiol. Biotechnol.* **2015**, *26*, 385–393, doi:10.4014/jmb.1509.09012.
30. Flores, O.; Retamales, J.; Núñez, M.; León, M.; Salinas, P.; Besoain, X.; Yañez, C.; Bastías, R. Characterization of Bacteriophages

against *Pseudomonas syringae* pv. *actinidiae* with Potential Use as Natural Antimicrobials in Kiwifruit Plants. *Microorganisms* **2020**, *8*, 1–17, doi:10.3390/microorganisms8070974.

31. Pinheiro, L.A.M.; Pereira, C.; Barreal, M.E.; Gallego, P.P.; Balcão, V.M.; Almeida, A. Use of Phage Φ6 to Inactivate *Pseudomonas syringae* pv. *actinidiae* in Kiwifruit Plants: In Vitro and Ex Vivo Experiments. *Appl. Microbiol. Biotechnol.* **2020**, *104*, 1319–1330, doi:10.1007/s00253-019-10301-7.
32. Ni, P.; Wang, L.; Deng, B.; Jiu, S.; Ma, C.; Zhang, C.; Almeida, A.; Wang, D.; Xu, W.; Wang, S. Combined Application of Bacteriophages and Carvacrol in the Control of Pseudomonas Syringae Pv. Actinidiae Planktonic and Biofilm Forms. *Microorganisms* **2020**, *8*, 837, doi:10.3390/microorganisms8060837.
33. Rabiey, M.; Roy, S.R.; Holtappels, D.; Franceschetti, L.; Quilty, B.J.; Creeth, R.; Sundin, G.W.; Wagemans, J.; Lavigne, R.; Jackson, R.W. Phage Biocontrol to Combat *Pseudomonas syringae* Pathogens Causing Disease in Cherry. *Microb. Biotechnol.* **2020**, *13*, 1428–1445, doi:10.1111/1751-7915.13585.
34. Rombouts, S.; Volckaert, A.; Venneman, S.; Declercq, B.; Vandenheuvel, D.; Allonsius, C.N.; Van Malderghem, C.; Jang, H.B.; Briers, Y.; Noben, J.P.; et al. Characterization of Novel Bacteriophages for Biocontrol of Bacterial Blight in Leek Caused by *Pseudomonas syringae* pv. *porri. Front. Microbiol.* **2016**, *7*, 178202, doi:10.3389/fmicb.2016.00279.
35. Boulé, J.; Sholberg, P.L.; Lehman, S.M.; O'Gorman, D.T.; Svircev, A.M. Isolation and Characterization of Eight Bacteriophages Infecting *Erwinia amylovora* and Their Potential as Biological Control Agents in British Columbia, Canada. *Can. J. Plant Pathol.* **2011**, *33*, 308–317, doi:10.1080/07060661.2011.588250.
36. Schwarczinger, I.; Kolozsváriné Nagy, J.; Künstler, A.; Szabó, L.; Geider, K.; Király, L.; Pogány, M. Characterization of Myoviridae and Podoviridae Family Bacteriophages of *Erwinia amylovora* from Hungary - Potential of Application in Biological Control of Fire Blight. *Eur. J. Plant Pathol.* **2017**, *149*, 639–652, doi:10.1007/s10658-017-1214-9.
37. Sabri, M.; El Handi, K.; Valentini, F.; De Stradis, A.; Achbani, E.H.; Benkirane, R.; Resch, G.; Elbeaino, T. Identification and Characterization of *Erwinia phage* IT22: A New Bacteriophage-Based Biocontrol against *Erwinia amylovora. Viruses* **2022**, *14*, 2455, doi:10.3390/v14112455.
38. Born, Y.; Fieseler, L.; Thöny, V.; Leimer, N.; Duffy, B.; Loessner, M.J. Engineering of Bacteriophages Y2::DpoL1-C and Y2::LuxAB for Efficient Control and Rapid Detection of the Fire Blight Pathogen, *Erwinia amylovora*. *Appl. Environ. Microbiol.* **2017**, *83*, doi:10.1128/AEM.00341-17.
39. Biosca, E.G.; Delgado Santander, R.; Morán, F.; Figàs-Segura, À.; Vázquez, R.; Català-Senent, J.F.; Álvarez, B. First European *Erwinia amylovora* Lytic Bacteriophage Cocktails Effective in the Host: Characterization and Prospects for Fire Blight Biocontrol. *Biology (Basel).* **2024**, *13*, 176, doi:10.3390/biology13030176.
40. Das, M.; Bhowmick, T.S.; Ahern, S.J.; Young, R.; Gonzalez, C.F. Control of Pierce's Disease by Phage. *PLoS One* **2015**, *10*, e0128902, doi:10.1371/journal.pone.0128902.
41. Bagchus, C.; Niederau, P.A.; Ceelen, M.; Balletbó Canals, A.; Hooftman, R.; Kuipers, B.; Castanedo Fontanillas, S.; Huijs, H.; Appelman, N.; Kuiper, S.; et al. Xylencer: Silencing *Xylella fastidiosa* Available online: https://2019.igem.org/Team:Wageningen_UR.

Disclaimer/Publisher's Note: The statements, opinions and data contained in all publications are solely those of the individual author(s) and contributor(s) and not of MDPI and/or the editor(s). MDPI and/or the editor(s) disclaim responsibility for any injury to people or property resulting from any ideas, methods, instructions or products referred to in the content.
