# Supplementary material for: Phage Therapy in Plant Disease Management: 110 Years of History, Current Challenges, and Future Trends
Source: Plants (Basel). 2026 Jan 24;15(3):368. doi: 10.3390/plants15030368 (PMC12899248; doi:10.3390/plants15030368)
Supplement: Supplementary file 1 [file plants-15-00368-s001.zip › Supplementary Material S3. Table S1. Overview of the in vivo efficacy of bacteriophages in different pathosystems.pdf]

Supplementary material

Phage Therapy in Plant Disease Management: 110 Years of History, Current Challenges, and Future Trends

Botond Zsombor Pertics, Lóránt Király, Zoltán Bozsó *, Dániel Krüzselyi, Judit Kolozsváriné Nagy, András Künstler, Ferenc Samu and Ildikó Schwarczinger *

Plant Protection Institute, Hungarian Research Network Centre for Agricultural Research, Fehérvári út 132–144, 1116 Budapest, Hungary; pertics.botond@atk.hun-ren.hu (B.Z.P.); kiraly.lorant@atk.hun-ren.hu (L.K.); kruzselyi.daniel@atk.hun-ren.hu (D.K.); nagy.judit@atk.hun-ren.hu (J.K.N.); kunstler.andras@atk.hun-ren.hu (A.K.); samu.ferenc@atk.hun-ren.hu (F.S.)

* Correspondence: bozso.zoltan@atk.hun-ren.hu (Z.B.); schwarczinger.ildiko@atk.hun-ren.hu (I.S.); Tel.: +36-30-486-2416 (Z.B.)

Table S1. Overview of the *in vivo* efficacy of bacteriophages in different pathosystems (2000-2025)

Host	Disease	Pathogen	Phage	PFU/mL	Application	Efficacy	Year	References
**potato**	soft rot	*Pectobacterium atrosepticum*	Nepra, Nobby, Slant, Lelidair, Gaspode and Momine	10^8	tuber treatments with phage cocktail under simulated storage conditions	The cocktail reduced both disease incidence and disease severity by 61% and 64%, respectively.	2019	[1]
**potato**	soft rot	*P. carotovorum* subsp. *carotovorum, P. wasabiae and Dickeya solani*	ΦPD10.3 and ΦPD23.1	10^5	application of individual or combined phages with the pathogens (co-inoculation)	Phages reduced soft rot severity caused by the three soft rot pathogens, by 80% in potato slices and by 95% in whole tubers.	2015	[2]
**potato**	blackleg and soft rot	*Dickeya solani*	φDs3CZ and φDs20CZ	10^6–10^8	preventive and curative seed tuber treatment	Better efficacy was achieved when a higher concentration of phage cocktail was used. Preventative application of phage cocktail resulted in a decrease in infection intensity of 86.7% and 54.6% in curative application during greenhouse experiments.	2024	[3]
**potato**	blackleg and soft rot	*D. solani*	vB_DsoM_LIMEstone1 and vB_DsoM_LIMEstone2	10^{10}	seed tuber treatment	The phages increased yield by 13% and reduced disease incidence by 5%.	2012	[4]
**potato**	common scab	*Streptomyces scabies*	phiAS1	10^9	diseased tubers were bathed in a phage suspension	Potato seed tubers treated with phage resulted in tuber progeny with scab surface lesion of 1.2% compared with tubers harvested from untreated seed tubers with 23%.	2001	[5]
**potato**	bacterial wilt	*Ralstonia solanacearum*	P1 cocktail (P-PSG-1, P-PSG-2, P-PSG-3,	10^9	injection of the phages into the plants or decontamination of sterilized soil	Phage cocktail protected 80% of potato plants from wilt and destroyed 98% of the target bacteria in the soil one week after spraying.	2017	[6]

			P-PSG-7, P-PSG-8, P-PSG-9)		spiked with R. solanacearum.			
**tomato & potato**	bacterial wilt	*R. solanacearum*	ϕsp1	10^8	soil drenching of infected tomato plants with phage; phage-treated potato tuber slices	Plant bioassays showed 81.39 and 87.75% reduction in pathogen counts using phage in potato tubers and in soil of tomato seedlings, respectively.	2021	[7]
**tomato**	bacterial wilt	*R. solanacearum*	φRSA1, φRSB1, and φRSL1.	10^6	pre-treatment of single phage on tomato plants	Phage application resulted no symptoms of bacterial wilt.	2011	[8]
**tomato**	bacterial wilt	*R. solanacearum*	RsPod1E GY	10^{11}	pretreatment of soil, in greenhouse/under greenhouse condition, tomato seedlings	Phage treatment resulted no disease symptoms.	2018	[9]
**tomato**	bacterial wilt	*R. solanacearum*	*Podovirida e*	10^6	in greenhouse and field experiments.	Phage cocktail application decreased the incidence of disease by up to 80%.	2019	[10]
**tomato**	bacterial wilt	*R. solanacearum*	vRsoP-WF2, vRsoP-WM2, and vRsoP-WR2	10^6–10^{10}	co-inoculation of pathogen and single phage or phage combinations by irrigation of soil	Phages significantly decreased bacterial wilt incidence in different biocontrol assays by up to 20–6%, compared to 80% in positive controls, Decreases in bacterial wilt percentages up to 3–0% were observed when mixtures of binary combinations and all the three phages were used.	2019	[11]
**tomato**	bacterial wilt	*R. solanacearum*	NJ-P3, NB-P21, NC-P34, and NN-P42,	10^5	greenhouse and field experiments, curative phage treatment	Single application of a four-phage cocktail significantly reduced disease incidence by 33–40%, while two- or three times application of phage cocktail significantly reduced incidence by 67–84%, as compared to 100% in control treatment.	2024	[12]

**tomato**	bacterial wilt	*R. pseudosolanacearum*	RpT1 and RpY2	10^6–10^8	three days post-treatment	Treatment of tomato plants with single phage or phage cocktail at 10^8 PFU/g soil significantly reduced disease severity up to 40% as compared to control with (80%).	2022	[13]
**tomato**	bacterial leaf spot	*Xanthomonas euvesicatoria*	AgriPhage™	10^{10}	greenhouse and field experiments	Phage cocktails formulated with pregelatinized corn fluor, sucrose; Casecrete or skim milk, could reduce disease severity and provide better protection to plants than unformulated phages.	2003	[14]
**pepper**	bacterial leaf spot	*X. euvesicatoria*	KΦ1	10^8	preventive or curative phage treatments, foliar spray of pepper plants, in greenhouse trials	Phages significantly reduced the lesion number on pepper leaves compared to the non-phage treated control. Efects of double applications (pre- and post-pathogen inoculation) were not statistically different from single variant treatments. The integration of the phage KΦ1 and copper-hydroxide treatments resulted in an increased efficacy compared to copper-hydroxide alone.	2018	[15]
**tomato & tobacco**	bacterial wilt	*R. solanacearum*	engineered 'Trojan horse' phage		co-inoculaton/ post treatment	"Trojan horse" to deliver a CRISPR-AsCas12f1 system to disarm the virulence gene hrpB in the pathogen effectively controlled bacterial wilt in both laboratory assays and plants.	2024	[16]
**cherry**	bacterial canker	*Pseudomonas syringae* pv. *syringae*	MR6, MR8, MR15 and MR16	10^8	co-inoculation (pathogen + single phage or phage cocktail), number of reisolated bacterial colonies were counted	Two days after inoculation the number of bacterial colonies was reduced by phage MR6 and MR15 by almost 80% and 100% respectively. MR16 reduced the bacterial population to almost zero by week five. 13- and 7-cocktail suppressed Pss population growth at week five by 50%.	2020	[17]

**pear & apple**	fire blight	*Erwinia amylovora*	ΦEa1337-26 and ΦEa2345-6	10^8–10^9	phage with carrier microorganism (Pantoea agglomerans Eh21-5)	ΦEa1337-26 + Eh21-5 and ΦEa2345-6 + Eh21-5 reduced infection of detached pear blossoms by 84 and 96% respectively. ΦEa2345-6 + Eh21-5, reduced the infection on apple flowers of potted apple trees by 56% and compared well with the antibiotic streptomycin.	2011	[18]
**pear**	fire blight	*E. amylovora*	EP-IT22	10^8	stem injection of one-year old pear plants with a single phage	There were no symptoms of fire blight, similar to antibiotic treatment.	2022	[19]
**loquat**	fire blight	*E. amylovora*	ϕ21, ϕ25	10^8	pre-treatment or co-inoculation (phage cocktails + pathogen) on detached loquat fruits	Preventative phage treatment was better, reduced disease severity up to 66.7–100%. Phage cocktails achieved better effects.	2024	[20]
**apple**	fire blight	*E. amylovora*	ΦEa21-4 and ΦEa31-3	10^8	blossom blight incidence in field trials	The tested phages significantly reduced flower spot and their effect was comparable to that of AgriPhage, with disease reduction ranging from 68.5 to 82.7%. UV protectors received special attention.	2024	[21]
**apple**	fire blight	*E. amylovora*	ϕEF1 and ϕEF2	10^7	preventative application on detached pears	Single phages resulted in smaller fire blight lesions as compared to untreated controls. The phage cocktail reduced necrosis more effectively than the single phage treatments.	2025	[22]
**apple**	fire blight	*E. amylovora*	engeneered Y2::dpoL1-C	3 x 10^8	post treatment of detached apple flowers	The genetically engineered Y2::dpoL1-C bacteriophage reduced the bacterial titers up to 81-95% due to the increased production of exopolysaccharide depolymerase.	2017	[23]
**kiwifruit**	bacterial blight	*P. syringae* pv. *actinidiae*	CHF1, CHF7, CHF19,	10^8	under greenhouse conditions on two-year old kiwifruit plants	Phage mixtures reduced bacterial load on kiwifruit leaves 24h post-infection with Psa by more than 75% in comparison to untreated plants.	2020	[24]

			and CHF21					
**banana**	Moko wilt	*R. solanacearum*	*Podoviridae*	10^7	under greenhouse conditions	Two-phage cocktails provided 100% protection of plants from Moko disease, while treatments with single phages still resulted in disease symptoms.	2020	[25]
**citrus**	Asiatic citrus canker	*X. citri* pv. *citri*	n/a	5×10^9	pre- and post-treatment, foliar spray, two-year-old Mexican lime plants	Phage application with acibenzolar-S-methyl (ASM) showed 82.1 to 86.1% reduction in disease incidence.	2017	[26]
**citrus**	Asiatic citrus canker and citrus bacterial spot	*X. citri* pv. *citri*, *X. euvesicatoria* pv. *citrumelonis*	CP2, ΦXac2005-1, ccΦ7, and ccΦ13	10^8–5×10^9	pre-treatment, grapefruit, orange plants,	In the case of Asiatic citrus canker phage treatment reduced disease severity by 59% compared to controls. The integration of phage and copper-mancozeb resulted in equal or less control than copper-mancozeb application alone. Phage treatment provided significant disease reduction (citrus bacterial spot) on moderately sensitive Valencia oranges in two trials (48 and 35%); however, on the highly susceptible grapefruit host it was ineffective.	2008	[27]
**grapevine**	Pierce's disease	*Xylella fastidiosa* subsp. *fastidiosa*	Sano, Salvo, Prado and Paz	10^{10}	pre- and post-treatment with phage cocktail, in greenhouse trials	Pierce's disease symptoms could be stopped using phage treatment post-infection as well as applying phage prophylactically. Post and pre-treatment with phage significantly reduced bacteria population (10 –1000-fold) compared to non-treated controls.	2015	[28]
**olive tree**	Pierce's disease	*X. fastidiosa* subsp. *fastidiosa*	XylPhi-PD™		bacterium, infected with genetically engineered phages, injected directly into vines	'XYLENCER project', engineered phages encode for a Pathogen Associated Molecular Pattern (PAMP) so that phage infection and bacterial lysis trigger plant immune responses. It reduced disease incidence by 57%.	2019	[29]

**mushroom**	brown blotch	*P. tolaasii*	ϕPto-bp6g	10^6	co-inoculation	Blotches on mushrooms were completely blocked by co-incubation with phage.	2012	[30]
**Chinese cabbage**	soft rot	*Pectobacterium spp.*	phiPccP-1, phiPccP-2 and phiPccP-3	10^8	Pre-treatment	The individual phage or the cocktail significantly reduced soft rot symptoms by 60–95% in detached mature leaves, and the phage cocktail had greater control efficacy, as high as commercial antibiotics. While pretreatments with individual phage or a phage cocktail on seedlings reduced symptom severity it was not as effective as antibiotics.	2024	[31]
**onion**	bacterial leaf blight	*X. axonopodis* pv. *allii*	AgriPhage™	10^5–10^8	plant defense activator, acibenzolar-S-methyl+phage, under field and greenhouse conditions	Phage applications weekly and biweekly in field trials reduced disease severity by 26 to 50%, similar to treatments of copper-mancozeb. Acibenzolar-S-methyl also successfully reduced disease severity by up to 50% when used alone preventatively or followed by biweekly bacteriophage applications.	2007	[32]
**onion**	soft rot	*P. carotovorum* subsp. *carotovorum*	φMA11, φMA12, φMA13 and φMA14	10^6–10^8	phage cocktail (immersion, and spraying), onions in field conditions	The cocktail of four phages significantly reduced soft rot frequency and yield after harvest as compared to untreated controls.	2020	[33]
**leek**	bacterial blight	*Pseudomonas syringae* pv. *porri (Psp)*	vB_PsyM_KIL1, vB_PsyM_KIL2, vB_PsyM_KIL3, and vB_PsyM_KIL3b.	10^7–10^8	field trials, pretreatments by submerging plants in the phage cocktail, post-treatment by spraying one day after *Psp* inoculation	Application of cocktail phage decreased disease incidence and disease severity but could not completely stop infection.	2016	[34]

**melon**	bacterial fruit blotch	*Acidovorax citrulli*	ACPWH	10^9	soil application	Phages reduced disease severity from 80% (of untreated controls) to 27%.	2020	[35]
**geranium**	bacterial blight	*X. campestris* pv. *pelargonii*	four h-mutant phages	5×10^8	seedling of geraniums under greenhouse conditions, curative application	Daily foliar sprays of four h-mutant phage mixture reduced disease incidence and severity by 50% and 75%, respectively. Daily phage treatment gave better results than weekly treatment.	2001	[36]
**broccoli**	black rot of crucifers	*X. campestris* pv. *campestris (Xcc)*	XcpSFC21 1 (*pXS*)	10^7	field trial, pre-treatment, application of phage with non-pathogenic *Xanthomonas* strain (*npX*), broccoli plants were sprayed with *npX* + *pXS*, then inoculated with Xcc.	Field experiments using combination of phage with the non-pathogenic *npX* strain resulted in a 59% disease incidence as compared the positive controls.	2017	[37]
**cauliflower**	black rot of crucifers	*X. campestris* pv. *campestris*	FoX2 and FoX6,	10^8–10^9	soil irrigation, treatment of cauliflower seedlings with phage cocktail	Preventative spray application of phage was more effective in reducing disease than co-inoculation. Pretreatment of the two-phage cocktail in field conditions led to a significant reduction in the number of symptomatic plants up to 67% compared to 96% in non-phage-treated plants.	2022	[38]
**kohlrabi**	black rot of crucifers	*X. campestris* pv. *campestris*	ϕXF1	10^7	single phage application of kohlrabi leaves	Phage treatment reduced disease symptoms, with black rot symptoms developing either late or not at all.	2025	[22]

References

1. Carstens, A.B.; Djurhuus, A.M.; Kot, W.; Hansen, L.H. A Novel Six-Phage Cocktail Reduces *Pectobacterium atrosepticum* Soft Rot Infection in Potato Tubers under Simulated Storage Conditions. *FEMS Microbiol. Lett.* **2019**, *366*, doi:10.1093/femsle/fnz101.
2. Czajkowski, R.; Ozymko, Z.; De Jager, V.; Siwinska, J.; Smolarska, A.; Ossowicki, A.; Narajczyk, M.; Lojkowska, E. Genomic, Proteomic and Morphological Characterization of Two Novel Broad Host Lytic Bacteriophages ΦPD10.3 and ΦPD23.1 Infecting Pectinolytic *Pectobacterium* spp. and *Dickeya* Spp. *PLoS One* **2015**, *10*, e0119812,

doi:10.1371/JOURNAL.PONE.0119812.
3. Kmoch, M.; Vacek, J.; Loubová, V.; Petrzik, K.; Brázdová, S.; Ševčík, R. Potential of Limestonevirus Bacteriophages for Ecological Control of *Dickeya solani* Causing Bacterial Potato Blackleg. *Agric.* **2024**, *14*, 497, doi:10.3390/agriculture14030497.
4. Adriaenssens, E.M.; van Vaerenbergh, J.; Vandenheuvel, D.; Dunon, V.; Ceyssens, P.J.; de Proft, M.; Kropinski, A.M.; Noben, J.P.; Maes, M.; Lavigne, R. T4-Related Bacteriophage LIMEstone Isolates for the Control of Soft Rot on Potato Caused by "*Dickeya solani*." *PLoS One* **2012**, *7*, e33227, doi:10.1371/journal.pone.0033227.
5. McKenna, F.; El-Tarabily, K.A.; Hardy, G.E.S.T.J.; Dell, B. Novel in Vivo Use of a Polyvalent *Streptomyces* Phage to Disinfest *Streptomyces scabies*-Infected Seed Potatoes. *Plant Pathol.* **2001**, *50*, 666–675, doi:10.1046/j.1365-3059.2001.00648.x.
6. Wei, C.; Liu, J.; Maina, A.N.; Mwaura, F.B.; Yu, J.; Yan, C.; Zhang, R.; Wei, H. Developing a Bacteriophage Cocktail for Biocontrol of Potato Bacterial Wilt. *Virol. Sin.* **2017**, *32*, 476–484, doi:10.1007/s12250-017-3987-6.
7. Umrao, P.D.; Kumar, V.; Kaistha, S.D. Biocontrol Potential of Bacteriophage ϕsp1 against Bacterial Wilt-Causing *Ralstonia solanacearum* in Solanaceae Crops. *Egypt. J. Biol. Pest Control* **2021**, *31*, 61-, doi:10.1186/s41938-021-00408-3.
8. Fujiwara, A.; Fujisawa, M.; Hamasaki, R.; Kawasaki, T.; Fujie, M.; Yamada, T. Biocontrol of *Ralstonia solanacearum* by Treatment with Lytic Bacteriophages. *Appl. Environ. Microbiol.* **2011**, *77*, 4155–4162, doi:10.1128/AEM.02847-10.
9. Elhalag, K.; Eldin, M.N.; Hussien, A.; Ahmad, A. Potential Use of Soilborne Lytic Podoviridae Phage as a Biocontrol Agent against *Ralstonia solanacearum*. *J. Basic Microbiol.* **2018**, *58*, 658–669, doi:10.1002/jobm.201800039.
10. Wang, X.; Wei, Z.; Yang, K.; Wang, J.; Jousset, A.; Xu, Y.; Shen, Q.; Friman, V.P. Phage Combination Therapies for Bacterial Wilt Disease in Tomato. *Nat. Biotechnol.* **2019**, *37*, 1513–1520, doi:10.1038/s41587-019-0328-3.
11. Álvarez, B.; López, M.M.; Biosca, E.G. Biocontrol of the Major Plant Pathogen Ralstonia Solanacearum in Irrigation Water and Host Plants by Novel Waterborne Lytic Bacteriophages. *Front. Microbiol.* **2019**, *10*, 492073, doi:10.3389/fmicb.2019.02813.
12. Wang, X.; Wang, S.; Huang, M.; He, Y.; Guo, S.; Yang, K.; Wang, N.; Sun, T.; Yang, H.; Yang, T.; et al. Phages Enhance Both Phytopathogen Density Control and Rhizosphere Microbiome Suppressiveness. *MBio* **2024**, *15*, doi:10.1128/mbio.03016-23.
13. Thapa Magar, R.; Lee, S.Y.; Kim, H.J.; Lee, S.W. Biocontrol of Bacterial Wilt in Tomato with a Cocktail of Lytic Bacteriophages. *Appl. Microbiol. Biotechnol.* **2022**, *106*, 3837–3848, doi:10.1007/s00253-022-11962-7.
14. Balogh, B.; Jones, J.B.; Momol, M.T.; Olson, S.M.; Obradovic, A.; King, P.; Jackson, L.E. Improved Efficacy of Newly Formulated Bacteriophages for Management of Bacterial Spot on Tomato. *Plant Dis.* **2003**, *87*, 949–954, doi:10.1094/PDIS.2003.87.8.949.
15. Gašić, K.; Kuzmanović, N.; Ivanović, M.; Prokić, A.; Šević, M.; Obradović, A. Complete Genome of the *Xanthomonas euvesicatoria* Specific Bacteriophage KΦ1, Its Survival and Potential in Control of Pepper Bacterial Spot. *Front. Microbiol.* **2018**, *9*, doi:10.3389/fmicb.2018.02021.
16. Peng, S.; Xu, Y.; Qu, H.; Nong, F.; Shu, F.; Yuan, G.; Ruan, L.; Zheng, D. Trojan Horse Virus Delivering CRISPR-AsCas12f1 Controls Plant Bacterial Wilt Caused by *Ralstonia solanacearum. MBio* **2024**, *15*, doi:10.1128/mbio.00619-24.
17. Rabiey, M.; Roy, S.R.; Holtappels, D.; Franceschetti, L.; Quilty, B.J.; Creeth, R.; Sundin, G.W.; Wagemans, J.; Lavigne, R.; Jackson, R.W. Phage Biocontrol to Combat *Pseudomonas* syringae Pathogens Causing Disease in Cherry. *Microb. Biotechnol.* **2020**, *13*, 1428–1445, doi:10.1111/1751-7915.13585.
18. Boulé, J.; Sholberg, P.L.; Lehman, S.M.; O'Gorman, D.T.; Svircev, A.M. Isolation and Characterization of Eight Bacteriophages Infecting *Erwinia amylovora* and Their Potential as

Biological Control Agents in British Columbia, Canada. *Can. J. Plant Pathol.* **2011**, *33*, 308–317, doi:10.1080/07060661.2011.588250.

19. Sabri, M.; El Handi, K.; Valentini, F.; De Stradis, A.; Achbani, E.H.; Benkirane, R.; Resch, G.; Elbeaino, T. Identification and Characterization of *Erwinia* Phage IT22: A New Bacteriophage-Based Biocontrol against *Erwinia amylovora*. *Viruses* **2022**, *14*, 2455, doi:10.3390/v14112455.
20. Biosca, E.G.; Delgado Santander, R.; Morán, F.; Figàs-Segura, À.; Vázquez, R.; Català-Senent, J.F.; Álvarez, B. First European *Erwinia amylovora* Lytic Bacteriophage Cocktails Effective in the Host: Characterization and Prospects for Fire Blight Biocontrol. *Biology (Basel).* **2024**, *13*, 176, doi:10.3390/biology13030176.
21. Gdanetz, K.; Dobbins, M.R.; Villani, S.M.; Outwater, C.A.; Slack, S.M.; Nesbitt, D.; Svircev, A.M.; Lauwers, E.M.; Zeng, Q.; Cox, K.D.; et al. Multisite Field Evaluation of Bacteriophages for Fire Blight Management: Incorporation of Ultraviolet Radiation Protectants and Impact on the Apple Flower Microbiome. *Phytopathology* **2024**, *114*, 1028–1038, doi:10.1094/PHYTO-04-23-0145-KC.
22. Vique, G.; Mendoza-Barberá, E.; Ramos-Barbero, M.D.; Blanco-Picazo, P.; Sala-Comorera, L.; Quirós, P.; Atares, S.; Salaet, I.; Muniesa, M.; Rodríguez-Rubio, L. Efficacy of *Erwinia amylovora* and *Xanthomonas campestris* pv *campestris* Phages to Control Fire Blight and Black Rot in Vivo . *Microbiol. Spectr.* **2025**, *13*, doi:10.1128/spectrum.00280-25.
23. Born, Y.; Fieseler, L.; Thöny, V.; Leimer, N.; Duffy, B.; Loessner, M.J. Engineering of Bacteriophages Y2::DpoL1-C and Y2::LuxAB for Efficient Control and Rapid Detection of the Fire Blight Pathogen, *Erwinia amylovora*. *Appl. Environ. Microbiol.* **2017**, *83*, doi:10.1128/AEM.00341-17.
24. Flores, O.; Retamales, J.; Núñez, M.; León, M.; Salinas, P.; Besoain, X.; Yañez, C.; Bastías, R. Characterization of Bacteriophages against *Pseudomonas syringae* pv. *actinidiae* with Potential Use as Natural Antimicrobials in Kiwifruit Plants. *Microorganisms* **2020**, *8*, 1–17, doi:10.3390/microorganisms8070974.
25. Ramírez, M.; Neuman, B.W.; Ramírez, C.A. Bacteriophages as Promising Agents for the Biological Control of Moko Disease (*Ralstonia solanacearum*) of Banana. *Biol. Control* **2020**, *149*, 104238, doi:10.1016/j.biocontrol.2020.104238.
26. Ibrahim, Y.E.; Saleh, A.A.; Al-Saleh, M.A. Management of Asiatic Citrus Canker under Field Conditions in Saudi Arabia Using Bacteriophages and Acibenzolar-s-Methyl. *Plant Dis.* **2017**, *101*, 761–765, doi:10.1094/PDIS-08-16-1213-RE.
27. Balogh, B.; Canteros, B.I.; Stall, R.E.; Jones, J.B. Control of Citrus Canker and Citrus Bacterial Spot with Bacteriophages. *Plant Dis.* **2008**, *92*, 1048–1052, doi:10.1094/PDIS-92-7-1048.
28. Das, M.; Bhowmick, T.S.; Ahern, S.J.; Young, R.; Gonzalez, C.F. Control of Pierce's Disease by Phage. *PLoS One* **2015**, *10*, e0128902, doi:10.1371/journal.pone.0128902.
29. Bagchus, C.; Niederau, P.A.; Ceelen, M.; Balletbó Canals, A.; Hooftman, R.; Kuipers, B.; Castanedo Fontanillas, S.; Huijs, H.; Appelman, N.; Kuiper, S.; et al. Xylencer: Silencing *Xylella fastidiosa* Available online: https://2019.igem.org/Team:Wageningen_UR.
30. Nguyen, H.T.D.; Yoon, S.; Kim, M.H.; Kim, Y.K.; Yoon, M.Y.; Cho, Y.H.; Lim, Y.; Shin, S.H.; Kim, D.E. Characterization of Bacteriophage ΦPto-Bp6g, a Novel Phage That Lyses *Pseudomonas tolaasii* Causing Brown Blotch Disease in Mushrooms. *J. Microbiol. Methods* **2012**, *91*, 514–519, doi:10.1016/j.mimet.2012.09.032.
31. Vu, N.T.; Kim, H.; Lee, S.; Hwang, I.S.; Kwon, C.T.; Oh, C.S. Bacteriophage Cocktail for Biocontrol of Soft Rot Disease Caused by *Pectobacterium* Species in Chinese Cabbage. *Appl. Microbiol. Biotechnol.* **2024**, *108*, 1–15, doi:10.1007/s00253-023-12881-x.
32. Lang, J.M.; Gent, D.H.; Schwartz, H.F. Management of *Xanthomonas* Leaf Blight of Onion with Bacteriophages and a Plant Activator. *Plant Dis.* **2007**, *91*, 871–878, doi:10.1094/PDIS-91-7-0871.
33. Zaczek-Moczydłowska, M.A.; Young, G.K.; Trudgett, J.; Fleming, C.C.; Campbell, K.; O'Hanlon, R. Genomic Characterization, Formulation and Efficacy in Planta of a Siphoviridae and Podoviridae Protection Cocktail against the Bacterial Plant Pathogens *Pectobacterium* spp. *Viruses* **2020**, *12*, 150, doi:10.3390/v12020150.
34. Rombouts, S.; Volckaert, A.; Venneman, S.; Declercq, B.; Vandenheuvel, D.; Allonsius, C.N.; Van Malderghem, C.; Jang, H.B.; Briers, Y.; Noben, J.P.; et al. Characterization of

Novel Bacteriophages for Biocontrol of Bacterial Blight in Leek Caused by *Pseudomonas syringae* pv. *porri*. *Front. Microbiol.* **2016**, *7*, 178202, doi:10.3389/fmicb.2016.00279.

35. Rahimi-Midani, A.; Choi, T.J. Transport of Phage in Melon Plants and Inhibition of Progression of Bacterial Fruit Blotch. *Viruses* **2020**, *12*, 477, doi:10.3390/v12040477.
36. Flaherty, J.E.; Harbaugh, B.K.; Jones, J.B.; Somodi, G.C.; Jackson, L.E. H-Mutant Bacteriophages as a Potential Biocontrol of Bacterial Blight of Geranium. *HortScience* **2001**, *36*, 98–100, doi:10.21273/hortsci.36.1.98.
37. Nagai, H.; Miyake, N.; Kato, S.; Maekawa, D.; Inoue, Y.; Takikawa, Y. Improved Control of Black Rot of Broccoli Caused by *Xanthomonas campestris* pv. *campestris* Using a Bacteriophage and a Nonpathogenic *Xanthomonas* sp. Strain. *J. Gen. Plant Pathol.* **2017**, *83*, 373–381, doi:10.1007/s10327-017-0745-4.
38. Holtappels, D.; Fortuna, K.J.; Moons, L.; Broeckaert, N.; Bäcker, L.E.; Venneman, S.; Rombouts, S.; Lippens, L.; Baeyen, S.; Pollet, S.; et al. The Potential of Bacteriophages to Control *Xanthomonas campestris* pv. *campestris* at Different Stages of Disease Development. *Microb. Biotechnol.* **2022**, *15*, 1762–1782, doi:10.1111/1751-7915.14004.

Disclaimer/Publisher's Note: The statements, opinions and data contained in all publications are solely those of the individual author(s) and contributor(s) and not of MDPI and/or the editor(s). MDPI and/or the editor(s) disclaim responsibility for any injury to people or property resulting from any ideas, methods, instructions or products referred to in the content.
